# Machine Learning-Driven Data Valuation for Optimizing High-Throughput Screening Pipelines

**DOI:** 10.1021/acs.jcim.4c01547

**Published:** 2024-10-23

**Authors:** Joshua Hesse, Davide Boldini, Stephan A. Sieber

**Affiliations:** Technical University of Munich, TUM School of Natural Sciences, Department of Bioscience, Center for Functional Protein Assemblies (CPA), 85748 Garching bei München, Germany

## Abstract

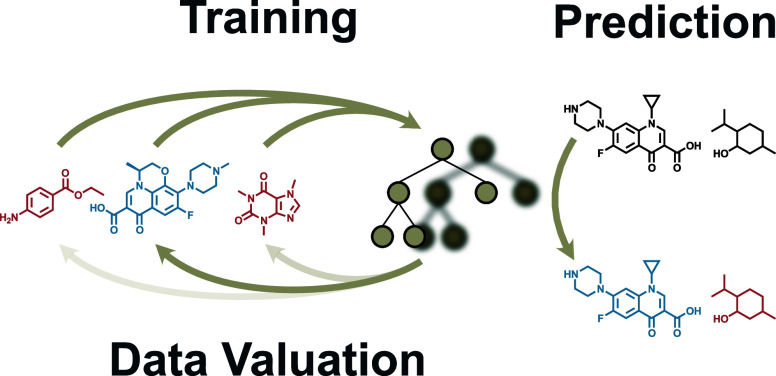

In the rapidly evolving field of drug discovery, high-throughput screening (HTS) is essential for identifying bioactive compounds. This study introduces a novel application of data valuation, a concept for evaluating the importance of data points based on their impact, to enhance drug discovery pipelines. Our approach improves active learning for compound library screening, robustly identifies true and false positives in HTS data, and identifies important inactive samples in an imbalanced HTS training, all while accounting for computational efficiency. We demonstrate that importance-based methods enable more effective batch screening, reducing the need for extensive HTS. Machine learning models accurately differentiate true biological activity from assay artifacts, streamlining the drug discovery process. Additionally, importance undersampling aids in HTS data set balancing, improving machine learning performance without omitting crucial inactive samples. These advancements could significantly enhance the efficiency and accuracy of drug development.

## Introduction

High-throughput screening (HTS) is a cornerstone in drug discovery, enabling the rapid testing of large chemical libraries for potential bioactivity.^1,2^ However, the efficiency of screening large numbers of molecules also introduces drawbacks during both laboratory procedures and subsequent data analysis.^3^ While HTS allows for the screening of large libraries without prior filtering, it often results in wasted resources and time, as most screened samples turn out to be inactive.^4−6^

Moreover, even among the samples identified as active during primary HTS, many are later found to be false positives. These problematic compounds can be detected due to various factors, including colloidal aggregation,^7^ autofluorescence,^8^ assay interference,^9,10^ chemical reactivity,^11^ measurement uncertainty,^12^ or instrument malfunction.^9,13,14^ Developing methods to identify false positives in primary HTS data is crucial to avoid unnecessary follow-up experiments on these compounds.^6,10,13^

To address these limitations in HTS pipelines, we introduce a variety of and machine learning (ML) and deep learning (DL) data valuation approaches to the field of drug development. Data valuation involves assigning a value or importance score to each piece of data based on its utility within a specific framework.^15^ This concept has gained prominence in recent years within machine learning, particularly in areas like federated learning, where it helps determine the significance of different data sets or data points in improving model performance.^16,17^ These methods enable the identification of valuable data sources and guide strategies for data selection and acquisition.^15^

Within the context of machine learning, data valuation methods estimate each training instance’s impact on the optimal performance of a ML or DL model.^18^ Some samples carry significant information that is beneficial to the model, while others provide little to no useful information, and some may even degrade model performance.^18,19^ Data valuation methods can compute these importance scores in a variety of ways, both algorithm-dependent and algorithm-agnostic. Some methods calculate the impact of a training sample on the prediction of a separate test set, while others score a training sample’s importance based on its own prediction.^19−21^ These importance scores have already been used for domain adaptation and to identify mislabeled training samples in other domains, such as tabular data, text, and image classification.^19,20,22^

However, they have rarely been applied to chemical applications. Here, these compound-specific importance scores can be utilized to guide screening efforts, identify false positives, and remove uninformative compounds, thereby addressing significant challenges in current HTS pipelines. Within this study, we apply data valuation to the following applications within the drug development pipeline:

- **Active Learning:** Active learning is a ML approach to screen large libraries stepwise and efficiently accumulate targets. Data valuation can aid in identifying interesting targets in large chemical compound collections, avoiding the need to screen the entire library.^23^
- **False and True Positive Identification:** After an initial HTS, data valuation methods can be applied to identify false and true positive samples without the need of predefined structural filters, reducing unnecessary time and costs in followup validation efforts.^13^
- **Importance Undersampling:** When using HTS data for followup ML applications, undersampling is often applied, as HTS results are usually highly imbalanced.^24^ Data valuation methods can aid in identifying important and unimportant samples within the majority class, allowing more targeted undersampling.

In this study, we benchmarked gradient boosting-based data valuation methods such as Minimal Variance Sampling Analysis (MVS-A) and CatBoost’s object importance, the Deep Neural Network (DNN)-based approach Tracing Gradient Descent (TracIn), Data Valuation using Reinforcement Learning (DVRL), and a K-nearest neighbor (KNN) approximation of Shapley Values (SVs).^13,19−22^

To evaluate these data valuation approaches for applications in active learning and false positive detection, we curated a selection of 25 HTS data sets varying in size, class imbalance, biological targets, assay technology, and false positive rates based on the Multifidelity PubChem Bioassay (MF-PCBA) benchmark.^25^ Additionally, to analyze the data valuation approach for undersampling, we utilized the MolData repository.^26^

## Results and Discussion

### Data Valuation Concepts

Data valuation assesses the impact of individual data points within a training data set.^27^ Not all data contribute equally to ML model performance; some significantly enhance accuracy, while others may introduce noise or biases.^19,28^ Identifying informative data points optimizes the training process, particularly when data acquisition is costly, and improves model robustness by mitigating the impact of harmful data.^20,27^

#### KNN Shapley Values

The first data valuation method is an approximation of the cooperative game theory concept of SVs. In ML, SVs are typically used to attribute the contribution of individual features to a model’s predictions.^29^ However, in the context of data valuation, SVs are calculated by determining the utility of a ML model for every possible subset of the training set with and without a sample of interest, then averaging the difference over all subsets.^30^ This shift from feature-based to data-based SVs allows for the assessment of each data point’s importance in the training set. However, this calculation is impractical for large data sets due to the exponential scaling of subset calculations. KNN SV applies the concept of SVs within the neighborhood of a validation set sample, reducing computational complexity by ignoring many subsets.^21,30^ In binary classification problems, minority class samples typically receive higher SVs, reflecting their greater impact on subset predictions. This is especially relevant for HTS data due to the high class imbalance. True positives within the active class tend to be more important than false positives, as their absence degrades test set predictions, while the absence of false positives does not.^21^

#### CatBoost Object Importance

The developers of Catboost implemented CatBoost Object Importance for calculating importance for test sample predictions called FastLeafInfluence. Based on the LeafInfluence method, it leverages a custom Leave-One-Out (LOO) retraining procedure to assess the importance of the excluded training sample. The algorithm approximates full model retraining by only adjusting the leaf weights of the model trained on the full data set, while keeping the tree splits unchanged.^22,31^ FastLeafInfluence scores for HTS data can be interpreted similarly to KNN SVs.

#### DVRL

By leveraging the reinforcement learning (RL) framework, DVRL computes importance values for training data.^32^ The data value estimator (agent) assigns sampling probabilities to training samples, which are then used by a predictor trained on these samples. The reward function evaluates the predictor on a separate validation set and provides feedback to the agent based on performance changes. Over time, the data value estimator prioritizes samples that maximize the reward, resulting in optimal importance scores.^19^ These scores can be interpreted analogously to the previous approaches.

#### TracIn

As a DL approach, TracIn tracks the importance of training samples on the loss change of a test sample during DNNs training.^20^ Ideally, TracIn would quantify the change in test sample loss when updating the weights for a single training sample. However, this is impractical, as gradient descent typically updates weights for groups of training samples. To address this, TracIn saves the model’s state at checkpoints and calculates importance scores as the dot product of loss gradients from training and test samples, weighted by the learning rate, and summed across all checkpoints.^20^ TracIn can also compute self-importance, measuring a training sample’s impact on its own prediction. In HTS data, active samples often have higher importance values than inactive ones due to their low prevalence. However, unlike previous methods, TracIn assigns higher importance values to false positives than to true positives. This occurs because false positives have large gradients on their own predictions, as their absence from the training set would likely lead them to be classified as inactive.^20^

#### MVS-A

Another Gradient Boosting Machine (GBM)-based data valuation method, MVS-A, calculates importance scores during training, similar to TracIn.^13^ Unlike TracIn, which traces gradient changes, MVS-A tracks changes in the decision tree structure. Each tree in a gradient boosting model serves as a checkpoint, with importance scores calculated by evaluating the loss function gradients and Hessians for each sample. The scores are similar to TracIn’s self-importance approach, where the loss function gradient and Hessian of each sample are multiplied by themselves, quantifying their impact on their own prediction. MVS-A importance scores can be interpreted analogously to those of TracIn.

### Active Learning

**Figure 1:**
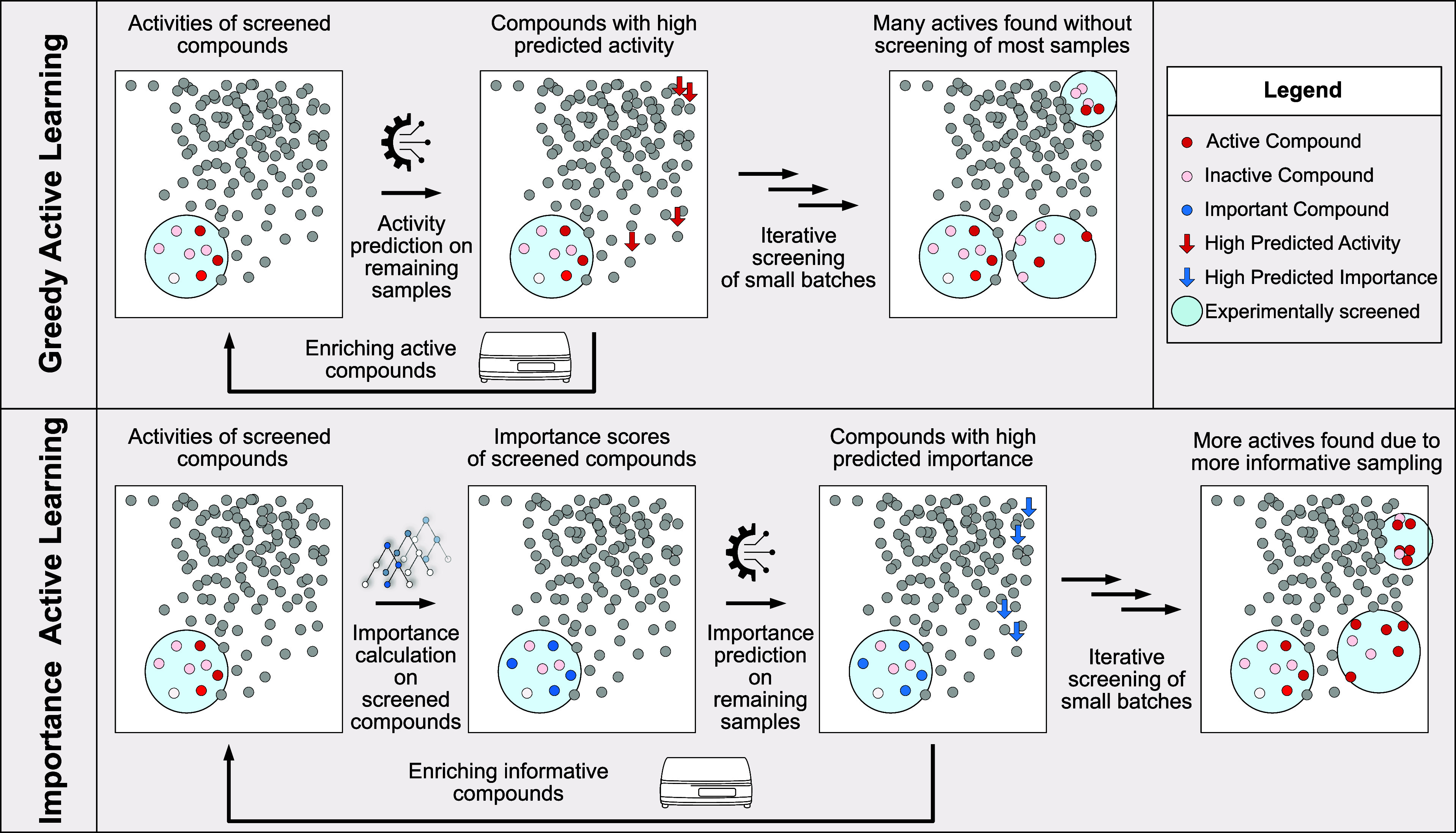
Active learning workflow. Greedy active learning: screen an initial batch of compounds, use activity scores to train an ML model, predict and validate promising candidates, and repeat to identify actives efficiently. Importance active learning: use initial batch to calculate importance scores, train an ML model to predict important samples, validate promising candidates, and repeat to identify actives through informative sampling.

Our study adopts an active learning approach for screening compound libraries, focusing on more efficiently identifying active compounds.^33^ Traditionally, active learning involves iteratively validating small batches and using a ML model to predict actives in the remaining library (Figure 1, upper panel).^23,34^ However, this method typically favors samples likely to be active, which can skew the model’s learning. To address this, we integrate data valuation techniques to identify important samples in each batch and employ a regression model to estimate the importance of the remaining library samples (Figure 1, lower panel). This strategy ensures the selection of both critical active and inactive samples for subsequent batches, fostering a balanced data set that enhances the model’s accuracy and understanding of class boundaries. This refined approach streamlines the discovery of active compounds and strengthens the overall learning process.

To evaluate the potential of this pipeline, we compared its ability to identify active samples against the state-of-the-art greedy approach, implemented using a GBM classifier. This comparison and subsequent optimization were performed on the *cysteine_protease* data set, which represents a mean across all data sets in terms of size and imbalance.

**Figure 2:**
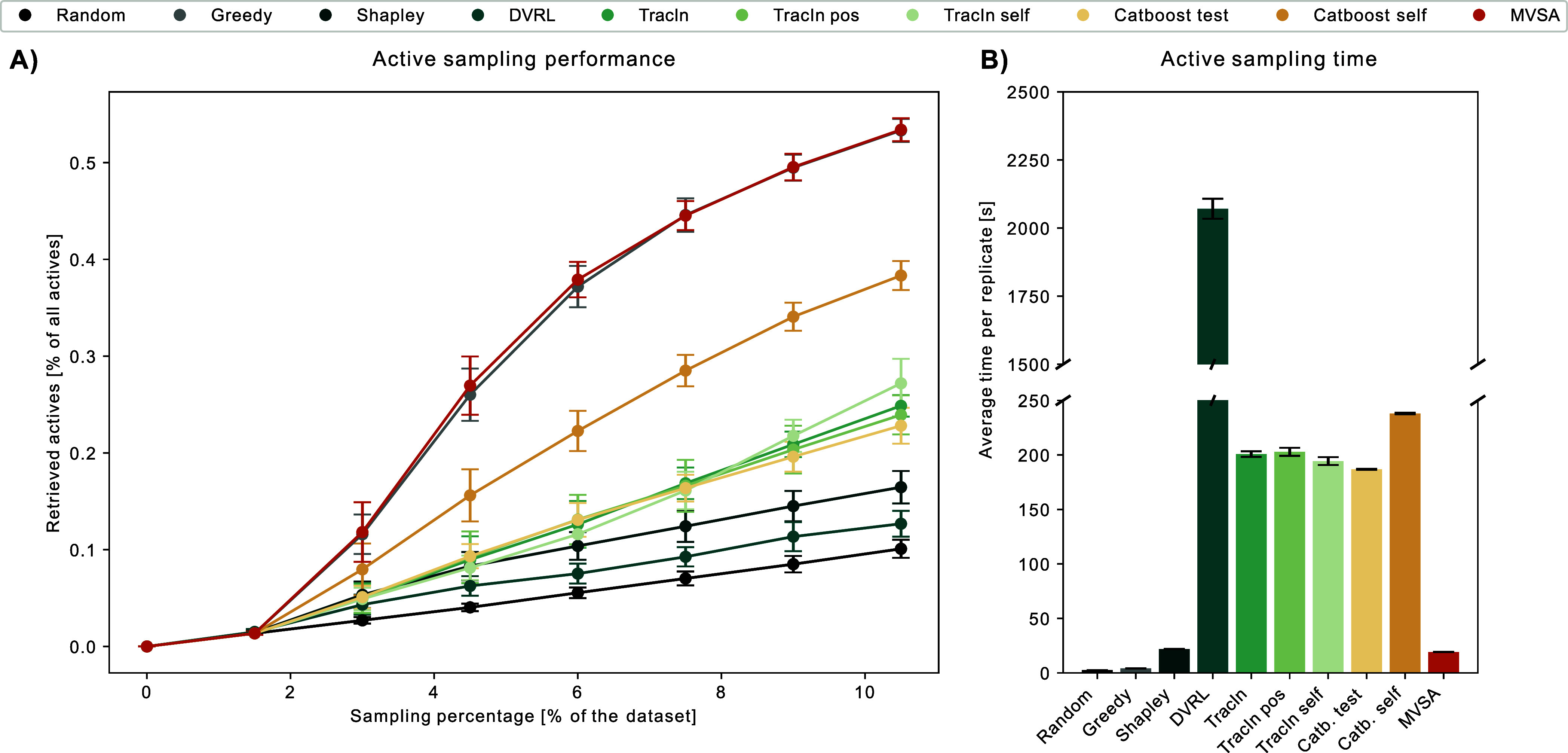
Active learning performance using data valuation methods: all calculations were done on the *cysteine_protease* data set, with 10 replicates per method. Each step size, including the initial random sampling, is 1.5% of the entire data set. A GBM was used for regression. (A) Percentage of actives found after each step. A Friedman test, a nonparametric test used to detect differences in performance across multiple methods, shows a highly significant difference (*P*-value = 1.07e–14), indicating that the methods do not perform equally. (B) Average time per replicate in seconds.

All active learning approaches significantly outperform the random sampling approach, confirming the advantage of ML-aided target selection (Figure S5A). Among them, MVS-A and greedy sampling significantly outperformed the other approaches, as seen in Figure 2.

Among them, DVRL came closest to random sampling. This result could be attributed to the small training set size or suboptimal model tuning, as DVRL involves two separate ML models with many parameters that require careful tuning. However, in practical applications, parameter tuning may not be feasible, as no clean training set would be available. Therefore, importance methods must perform well with minimal optimization to be applicable to any HTS data.

KNN SVs also performed similarly to DVRL, likely due to the limitations of the KNN algorithm when working with a small subset of the compound library, as KNN algorithms generally perform poorly at predictions outside their training domain.

TracIn performed slightly better than KNN SV, but was still significantly outperformed by MVS-A and CatBoost Figure S5A). This is likely due to differences in the underlying ML models. While TracIn uses DNNs to identify important samples, MVS-A and CatBoost use gradient boosting algorithms, which tend to perform better with smaller initial training sets. The discrepancy between MVS-A and CatBoost likely arises from differences in how they compute importance within gradient boosted trees. These results suggest that analyzing splitting decisions provides more accurate importance scores compared to leaf weight analysis.

While MVS-A performed on par with greedy sampling, we hypothesized that the bottleneck for MVS-A could be the importance score prediction of the regression model.

Consequently, we tested various regression models in the importance active learning pipeline on the same data set, consistently using MVS-A for importance calculation. Among these models, the sklearn implementation of Gaussian process regressor (GPR) showed the best active learning performance, improving the overall pipeline (Figure S3).

**Figure 3:**
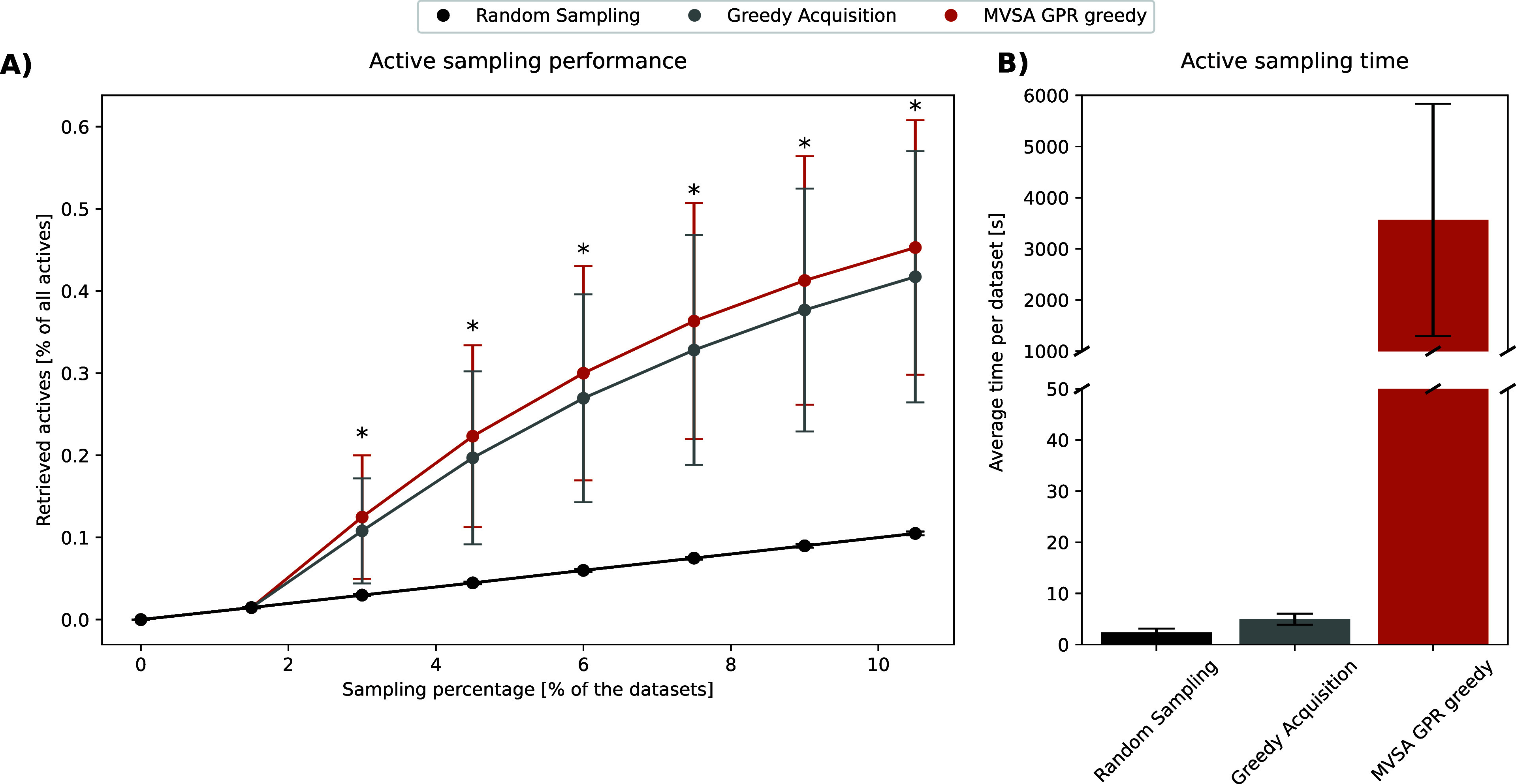
Importance-based active learning performance across all data sets. (A) Distribution of retrieved actives across all data sets. Each step size, including the initial random sampling, is 1.5% of the entire data set. Asterisks indicate that MVSA GPR greedy significantly outperformed the greedy benchmark (α = 0.05, 24 data sets) according to a Wilcoxon two-tailed single-rank test. (B) Average time taken per method across all 24 data sets.^35^

Finally, we benchmarked the optimized pipeline against the state-of-the-art greedy method on the remaining 24 data sets. As shown in Figure 3, the importance-based active learning approach significantly outperformed the greedy benchmark at all steps across the data set group.

This performance gain is likely due to the importance-based method sampling a more diverse set of compounds, including important inactive samples, thereby improving the model’s generalization. However, it is noteworthy that the state-of-the-art approach surpasses the importance-based method in computational speed, primarily due to the use of GPR. As shown in Figure 2, when coupled with a faster regression model like LightGBM, the importance-based approach performs comparably to the greedy approach.

These results are highly promising, showcasing the potential of importance-based methods to enhance active learning strategies. They offer a valuable pathway for researchers aiming to streamline their drug discovery pipelines.

### False Positive Identification

**Figure 4:**
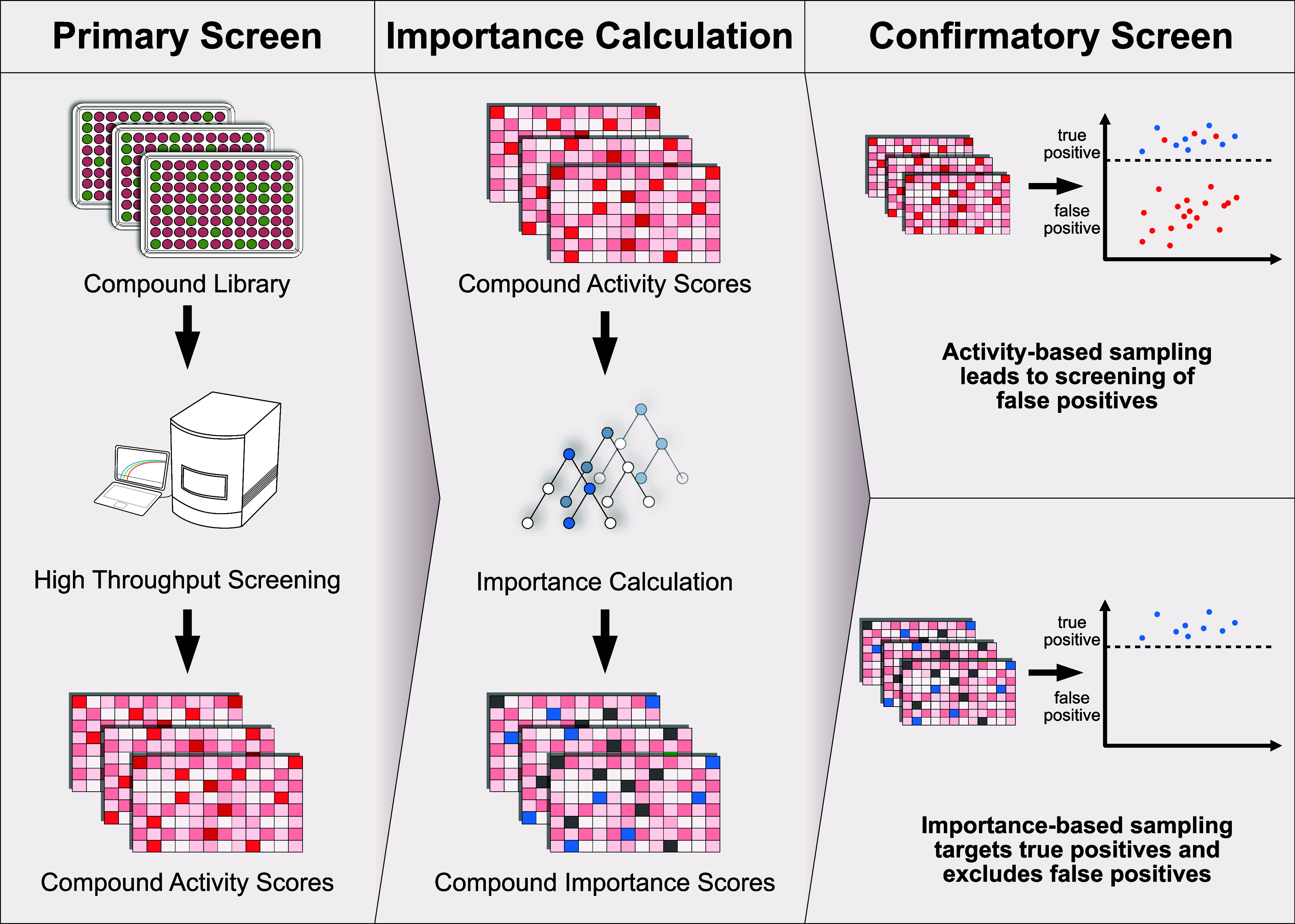
False and true positive detection workflow: a compound library is screened via HTS, resulting in activity scores. The activity scores can be used to train a ML model and calculate importance scores for each compound. Selecting compounds from the initial library based on their HTS activity often results in many false positives. In contrast, selecting samples according to their importance scores allows for more accurate sampling of true positives.

**Figure 5:**
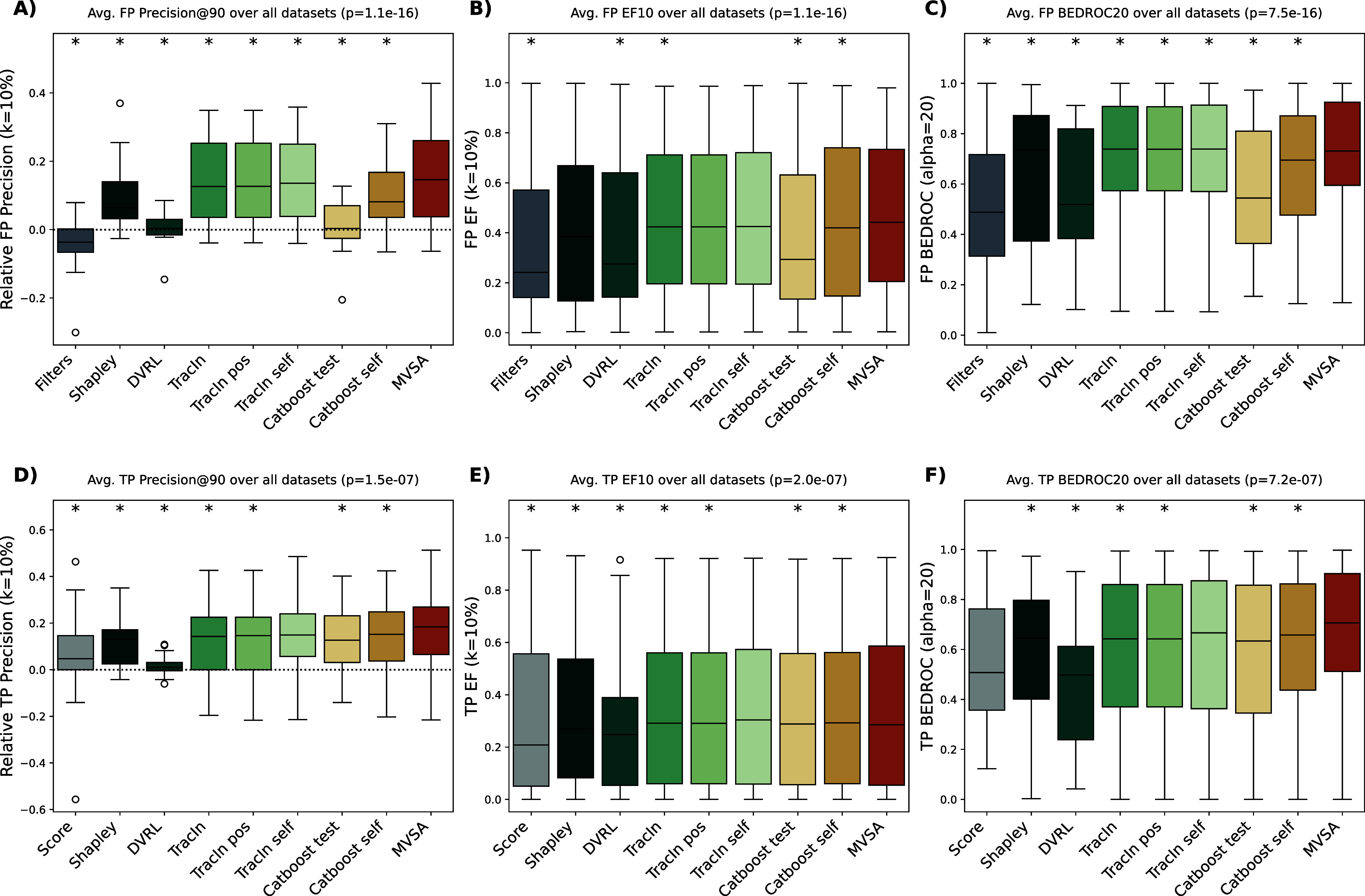
False and true positive detection performance across all data sets. (A–C) False positive performance metrics distributed over 25 data sets; asterisks indicate that the results are significantly different from MVS-A (α < 0.05, *n* = 10) according to a Wilcoxon two-tailed single-rank test with Benjamini Hochberg correction.^35,37,38^ (D–F) True positive performance metrics distributed over 25 data sets; asterisks indicate that the results are significantly different from MVS-A (α < 0.05, *n* = 10) according to a Wilcoxon two-tailed single-rank test with Benjamini Hochberg correction.^35,37,38^ (A) Relative false positive precision across all data sets; dotted line represents random sorting performance. (B) Mean false positive enrichment factor across all data sets. (C) Mean false positive BEDROC across all data sets. (D) Relative true positive precision across all data sets; dotted line represents random sorting performance. (E) Mean true positive enrichment factor across all data sets. (F) Mean true positive BEDROC across all data sets. *P*-values in the titles refer to Friedman test on all methods and data sets.^35^ All *P*-values are highly significant, indicating the methods do not perform equally according to all metrics presented.

Next, we applied the data valuation methods to the challenge of distinguishing between false and true positives. Using an HTS data set, each data valuation method was trained exclusively on the primary screening data (Figure 4). To evaluate their effectiveness in separating true from false positives, we compared the resulting importance rankings against the confirmatory screen data. Performance was measured using relative top-10 precision, enrichment factor, and Boltzmann-Enhanced Discrimination of the Receiver Operating Characteristic (BEDROC) scores. We performed a Friedman test across all data sets for each metric, with the resulting *P*-values displayed in Figure 5.

MVS-A significantly outperformed all other methods in false positive detection across two out of three metrics, with TracIn self and TracIn pos matching MVS-A’s performance on the enrichment factor score (Figure 5, Figure S13).

GSK and REOS structural filters performed significantly lower than all data valuation approaches in predicting false positives in two out of three metrics (Figure S13). However, it is important to note that GSK and REOS structural filters target not only false positives but also promiscuous compounds and those with undesirable pharmacological properties.^13,36^ These compounds may still show initial bioactivity, leading to lower performance in confirmatory screens. GSK and REOS structural filters also partially rely on a predefined set of fragments, which limits their identification of structurally diverse false positives. This can also be seen in Figure S6A, where the filters approach shows the lowest distribution of structural diversity in false positive detection. These two factors might explain the poor performance on this benchmark focused on primary assay false positives in a diverse chemical space.

KNN SV demonstrated a slight improvement in relative precision over random sorting. Notably, KNN SV performed best in relative precision for 6 out of 25 data sets (Figure S7).

KNN SV is the only data valuation method that outperforms random sorting in relative precision using only the validation set for importance prediction. This is likely due to the small number of neighbors in this approach. False positives generally have a negative impact on the prediction of inactive samples, as they might wrongly suggest activity for a given structure. For methods that use a large number of samples, such as DVRL or CatBoost, the impact of a single false positive on inactive sample prediction is minimal. However, with KNN, a false positive significantly influences the classification due to the small number of neighbors, making it more impactful. This effect is especially pronounced in highly imbalanced data sets, where the presence of even one active sample in a neighborhood can suggest activity for a new sample. Removing the false positive from that neighborhood positively impacts the prediction of inactive samples, giving it a larger negative importance than in models where the impact is diluted by many correctly labeled inactives. Additionally, KNN SV outperforms most other methods in terms of computation time (Figure S12).

TracIn-based methods performed well, being the only methods that matched MVS-A in one metric. Although TracIn methods excelled in only three data sets regarding relative precision, they consistently ranked near the top across all metrics. Interestingly, the performance between all three TracIn approaches was very similar. This was expected for TracIn and TracIn pos, as their only difference is the exclusion of inactive samples from the validation set. However, it was surprising that TracIn self performed equally well. This result likely stems from the fact that all TracIn approaches use self-prediction gradients, with TracIn self squaring these gradients, while the other approaches multiply them with gradients from the validation set. If most of the importance score is derived from the self-gradients, both methods yield similar outcomes.

TracIn self is particularly effective because it examines the effect of removing a training sample on its own prediction. As previously discussed, false positive sample gradients on their own prediction are very high, which explains why all TracIn methods perform similarly, with self-importance gradients dominating the importance scores.

This finding is especially promising, as it enables importance calculation—and, thus, false positive prediction—without needing a validation set. In practical applications, this allows for false positive detection directly on primary HTS data without first validating a subset in the laboratory.

CatBoost, unlike TracIn, showed a substantial difference in performance between CatBoost-self and CatBoost-test across all metrics. Notably, CatBoost-test performed worse than random sorting in almost half of the data sets (Figure S7A), while CatBoost-self almost always scored above 0.0. This poor performance of CatBoost-test is likely due to the same issues affecting KNN SV and DVRL, where the importance for inactive prediction is too small to detect. However, CatBoost-self allows for false positive prediction with similar performance to KNN SV, but without needing a validation set. This may be because removing false positives near activity cliffs enhances prediction performance for correctly labeled active samples, resulting in low importance scores for false positives in the CatBoost-self approach. This effect may be lost in noise when calculating the importance of a false positive on all samples, which is why it is only detectable via CatBoost-self and not with CatBoost-test. While CatBoost-self has a longer computational time than KNN SV (Figure S12), it significantly outperforms TracIn due to its use of gradient boosting instead of Feedforward Neural Networks (FNNs).

MVS-A also identifies the most diverse set of false positives, as shown in Figure S6A, followed by the TracIn methods. These results further confirm that self-importance based methods identify false positives by their uniqueness, rather than any common features.

MVS-A shows the best overall false positive prediction performance across all metrics, particularly in relative precision, where it outperforms all other methods in 13 out of 25 data sets (Figure S7). However, TracIn and MVS-A generally perform similarly, as they conceptually measure the same effect using different implementations. Both methods assess the impact of a given training sample on its own prediction, which is effective for false positive detection for reasons previously explained. MVS-A has a slight edge over TracIn in performance, likely because detecting changes in tree structure allows for more accurate importance score assignments compared to gradient changes in the last two layers of an FNN.

The results shown in Figure 5 were calculated using Extended-Connectivity Fingerprints (ECFPs) as the molecular representation. Experiments were also repeated using RDKit descriptors and, for TracIn, SMILES strings as chemical representations (Figures S8–S11).

Overall, most methods significantly outperformed the state-of-the-art fragment filter approach and showed substantial improvements in false positive prediction over random sorting, with MVS-A delivering the best overall performance.

### True Positive Identification

While false positives were identified by their high self-importance or low importance for the predictions of other samples, true positives can be detected using the opposite approach. True positives typically have a strong importance for the prediction of other samples because the ML model relies on them to recognize patterns. However, their self-importance is lower than that of false positives, as their activity can often be inferred from other true positives, reducing the dependency on their own presence in the training set.

Ranking actives according to their PubChem activity score is used as a benchmark method for true positive detection. Similar to false positive precision, the true positive precision is displayed as relative precision, where the true positive rates were subtracted from the precision scores prior to plotting.

The Score method performs slightly better than random sorting in regards to relative precision across all data sets as seen in Figure 5D. Figure S7D shows that the Score method actually outperforms all other methods on 5 out of 25 data sets, suggesting that the approach is viable given certain assay setups. The Score method actually performs best regarding structural diversity as seen in Figure S6B, which makes sense given the method is structure-agnostic and only uses the activity scores.

KNN SV significantly outperforms the Score method and comes close to TracIn, CatBoost, and MVS-A. This might be due to true positive samples having a large positive importance for the prediction of other true positive samples due to the strong imbalance in the data set. Additionally, within the active class, false positive samples should be ranked at the bottom according to importance, resulting in the top 10 percentile containing true positives.

DVRL, as with false positives, does not achieve significant performance above random sorting. Unlike in false positive detection, DVRL does not perform similarly to the CatBoost method, indicating that its complexity limits its applicability to this problem.

TracIn-self performs best out of the three TracIn approaches, with a more considerable difference to the other two TracIn versions compared to false positive prediction, showing the best relative precision performance for 5 out of 25 data sets (Figure S7D). These results are likely caused by the fact that the minority class will have larger self-importance values than the majority class, as removing one sample from the minority class will have a much bigger effect due to the lower number of remaining active samples used for classification. This leads to all actives having larger importance scores than inactives. Within the active class, the false positives cluster toward the largest self-importance scores for reasons explained previously. As a result, the low-importance actives tend to be true positives. This effect, that true positive detection is more of an indirect identification via the exclusion of false positives, might also be the reason why the overall performance of all data valuation methods decreases with regard to enrichment factor and BEDROC score, as can be seen when comparing Figure 5B,C and E,F. Remarkably, both approaches utilizing CatBoost exhibit similar levels of performance, which are on par with TracIn. This observation is especially interesting for CatBoost-test, which underperformed for false positive relative precision. This is likely because the importance of true positives on predicting other true positives is more easily detectable than the effect of false positives on the classification of inactives due to the strong imbalance of HTS data sets.

MVS-A once again outperforms all other methods on average across all data sets, showing the best relative precision in 13 out of 25 data sets. This success is likely due to its effectiveness in identifying false positives, which indirectly aids in identifying true positives. However, MVS-A performs worst regarding structural diversity in true positive predictions. This is expected, as MVS-A basically identifies highly unique compounds as false positives, and thereby indirectly identifies actives with similar structures are true positives.

### Importance Undersampling

To assess the impact of importance-based undersampling, we used the aging subset of the MolData benchmark for prediction performance measurements, ensuring comparability with existing benchmarks in the field. A LightGBM (LGBM) classifier was trained on each training set and validated on the respective validation set, with the process repeated iteratively as the training set was gradually reduced.

**Figure 6:**
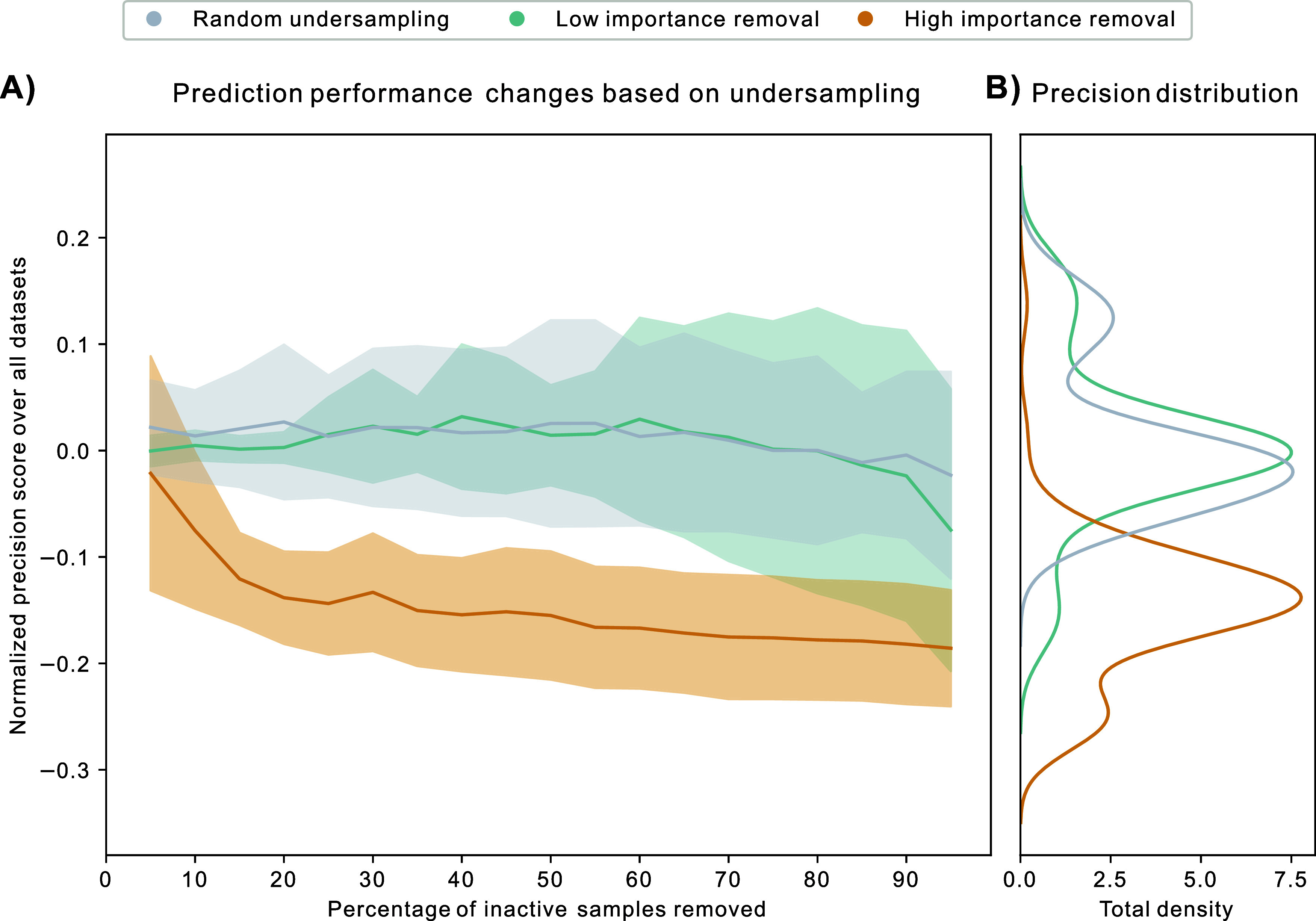
Undersampling performance of importance-based and random undersampling. (A) Average precision score performance of an LGBM classifier dependent on the undersampling strategy, averaged across aging data sets *vitamin_receptor*, *TNAP*, *TNAP_phosp*, and *HTRA*, with 10 replicates each; random and importance-based undersampling performance measured after every 5% step of undersampling. (B) Distribution of normalized precision scores across all data sets and all time points for each undersampling strategy.

At each iteration, 5% of the original training set was removed, either randomly or by removing inactive samples based on their importance scores. This iterative process continued until 95% of the training set was removed. Given MVS-A’s superior performance in previous experiments, it was selected as the data valuation method. Samples were ranked by importance, and at each step, either the most or least important inactive samples were removed. Although the aging data set group contains 10 data sets, Figure 6 focuses on the *vitamin_receptor*, *TNAP*, *TNAP_phosp*, and *HTRA* data sets. Figure S16 provides the difference in normalized precision scores across all 10 data sets. The remaining 6 data sets had low base prediction performance, resulting in uninformative importance scores, so we concentrated on data sets where the base predictor scored above 10% precision to generate meaningful results (Table S5).

Removing highly important inactives causes the model’s performance to deteriorate quickly, as seen in Figure 6A. As the most important samples are removed from the training set, the model loses the samples it learned most from, resulting in decreased performance. Figure 6B shows a clear shift in performance across all data sets and time points based on the undersampling method.

Removing low importance samples, on the other hand, does not decrease performance but instead increases it until a certain point, similar to random undersampling. This is due by the fact that removing unimportant samples reduces the imbalance of the data set, thereby lowering its bias to the majority class, without worsening its prediction performance, as these samples did not help the model learn its task.

To summarize, MVS-A was able to identify important inactive samples within the training sets, with their removal resulting in a strong decrease in classification performance. Interestingly, removing unimportant samples did not outperform random undersampling contrary to initial expectations. These results imply that MVS-A cannot differentiate between moderately important and irrelevant samples. However, importance undersampling could still be used to ensure the presence of important samples by augmenting random undersampling. Furthermore, exploring the concept of important inactive samples could provide deeper insights into class boundaries, potentially shedding light on activity cliffs within the chemical landscape of the given data set.

## Conclusions

This study introduced data valuation methods to various stages of the drug development pipeline, aiming to enhance current practices. The applications included improving active learning for more efficient chemical library screening, identifying false and true positive hits in primary HTS data, and optimizing HTS data for machine learning through importance undersampling.

The active learning results demonstrated that data valuation methods enable more efficient batch screening of large chemical libraries, reducing the need for extensive HTS screens to identify most actives. These findings, along with the importance undersampling results, suggest that sampling both actives and key inactives enhances machine learning models, leading to more efficient library screening. Although the importance-based method requires more computational time than the state-of-the-art approach, it identifies more actives with the same laboratory effort and resources, justifying the extra computation time.

In false positive detection, data valuation methods like MVS-A and TracIn significantly outperformed the current state-of-the-art fragment filter approach and random sorting, offering promising alternatives. Similarly, these methods competed with or outperformed the benchmark for true positive prediction, which sorts samples by activity.

The undersampling section demonstrated that data valuation methods can effectively identify important inactive samples in a highly imbalanced data set. These methods could enhance random undersampling by ensuring that crucial samples are retained in the reduced training set. Furthermore, analyzing these important inactive samples alongside the active class could provide valuable insights into the class boundaries, potentially highlighting the activity cliffs associated with the respective assay.

Overall, this study provided a first exploration of data valuation methods applied to chemical data, yielding encouraging results across all applications. These findings should inspire future research to further explore data valuation methods for optimizing HTS pipelines. To support this endeavor, we developed a toolkit for applying data valuation methods to HTS data, which can be accessed and expanded upon through our GitHub repository (https://github.com/JoshuaHesse/DataValuationPlatform).

## Methods

### Data Curation

Two groups of data sets are used. A curated set of 25 publicly available data sets is utilized for false positive detection and active learning, primarily based on the MF-PCBA benchmark. All 25 data sets consist of a primary high-throughput screen and a confirmatory screen on a subsection of the primary actives (Tables S1–S3). As some data valuation functions need a separate validation set to calculate importance values, ten percent with the highest primary screen PubChem activity scores were used as a separate validation set. To benchmark importance-driven undersampling, a subset of the MolData benchmark, a group of 10 data sets associated with the disease aging, was used (Table S4). Here, the train-test-val splits suggested by the MolData paper were kept. For all data sets, the SMILES strings were imported from the PubChem data sets, sanitized, and standardized into their canonical form using RDKit.^39^

### Molecular Representation

When used as input for LSTM calculations, SMILES were tokenized and vectorized using one-hot encoding, with the maximum length of each vector set to 120. RDKit was used to calculate the ECFPs for each molecule, with the radius set to two and the number of bits set to 1024.^39^ RDKit was also used to calculate a collection of 208 RDKit descriptors for each molecule.^39^

### Experimental Setup

#### Active Learning

In this experiment, data sets were iteratively screened to identify actives. The data valuation approaches use activity prediction followed by importance calculation on the training set and subsequent importance prediction on the remaining data set with a secondary regression model, a Gaussian process regressor. The remaining data set samples predicted to be most important by the regressor are sampled for the next iteration. For each data set, an initial training set comprising 1.5% of the entire data set was created using a stratified sampling approach from Scikit-learn’s train-test split function, ensuring that the ratio of active to inactive samples was preserved.^40^ This initial training set was then used to predict the next 1.5% of the data set for sampling. These newly sampled instances were added to the training set using their primary screen labels, and the next iteration of prediction was trained on the updated training set. After each iteration, the number of active samples identified was counted for each method to calculate the percentage of total actives found per step.

The benchmark method, called “greedy method”, uses direct activity prediction of the remaining library using an LGBM classifier. The classifier is trained on the training set and used to predict the remaining data set. The 1.5% of samples with the highest predicted probability were sampled.

Random sampling was used as a baseline method. At each step, the indices of the remaining data set were shuffled, and the first 1.5% were sampled

#### False and True Positive Prediction

For each data set, the data valuation approaches were trained on all samples minus the separate validation set. The importance scores were calculated and ranked, and the top and bottom 10% of all actives were identified as true and false positives, or vice versa depending on the method used. For all compounds within these top and bottom 10% that also have a confirmatory label, their prediction was analyzed using the precision score, enrichment factor, and BEDROC score.

For false positive prediction the GSK REOS filters were used as a benchmark using RDKit, ranking samples according to the number of applicable filters, with more filters indicating a higher likelihood of being a false positive.^39^ For true positive prediction, the Score method was used as a benchmark, ranking the samples according to their primary assay PubChem activity score. The PubChem activity score is a normalized score between 0 and 100, where the most active results of a bioassay are set close to 100 and the most inactive results close to 0. Some bioassays did not report PubChem activity scores in this manner. For these, alternative reported bioassay metrics were used and normalized to values between 0 and 100 to allow continuous ranking by the bioassay score. The Pubchem bioassay metrics used for the Score method are reported in Figure S2.

Random sorting was used as a baseline for both experiments regarding top-10 precision. Random sorting was defined as the false positive rate of each respective data set for false positive identification, and conversely, the true positive rate for true positive identification.

#### Importance Undersampling

Initially, for each MolData data set the importance scores were calculated for all training set samples. Then, an LGBM classifier was trained on the training set and validated on the validation set. The training set was then reduced by 5% of the original training set size by sorting according to the MVS-A importance scores and removing the highest or lowest 5% of inactives. The LGBM classifier was then trained and evaluated on the reduced training sets and the performance was calculated using Scikit-learn’s average precision score.^40^

As a baseline, random undersampling was applied instead of importance undersampling. Here, the indices of all inactives where randomly shuffled instead of sorted by importance. The first 5% of inactive sample indices were removed in each iteration.

### Model Development

#### KNN SVs

KNN SVs was implemented using the Datascope package.^21^ As this implementation uses K-nearest neighbor models to approximate Shapley values, which do not scale well with large and high dimensional data sets, a RandomUnderSampler from the imbalanced-learn package was used for undersampling, with a sampling strategy of 0.2, and a random seed according to the current replicate number.

#### CatBoost Object Importance

CatBoost Object Importance was implemented using a CatBoost model with 100 iterations and a random seed according to the current replicate was used. The model was trained on the training set. The training sample importance for the external validation set called test-importance, was calculated, as well as the importance for all active training samples, which will be called self-importance.

#### DVRL

DVRL was implemented using the codebase from Yoon et al.^19^ The DVRL approach contains two prediction models: the data value estimator and the predictor. As the predictor, an LGBMClassifier with default parameters and a random seed according to the index of the current replicate was used. The data value estimator is implemented as a deep neural network, which depends on the parameters of the DVRL object. The DVRL object was initialized by setting the hidden dimensions to 100, the combined dimensions to 10, and the iterations to 1000. A Rectified Linear Unit (ReLU) function was used for activation, the learning rate was set to 0.01, and the batch size was set to 5000. The DVRL object was trained using the ROC-AUC-score as the performance measurement for the predictor, which was measured on the external validation set.

#### TracIn

TracIn was implemented by adapting the code from Pruthi et al.^20^ TracIn is applied to a FNN implemented using tensorflow.^41^ A one-hidden-layer TensorFlow Long Short-Term Memory (LSTM) model was used instead when using SMILES strings as input (Table 1). Self-importance was implemented by squaring the loss gradients resulting from the change in the last two layers’ weights of the model checkpoint at 30, 60, and 90 epochs during training and summing over all 3 checkpoints. Test-importance was implemented by multiplying the loss gradients with all validation set gradients, averaging over all validation samples for each training sample and summing over all three checkpoints.

**Table 1:** Summary of TracIn Model Architectures

component	FNN model	LSTM model
input layer	dense, 512 neurons	LSTM, 64 units
input shape	(features)	(max_token_length, charset_length)
activation (input layer)	ELU	tanh
dropout (input layer)		0.2
hidden layer 1	dense, 512 neurons, ELU, Dropout 0.5	dense, 512 neurons, ELU, Dropout 0.5
hidden layer 2	dense, 512 neurons, ELU, Dropout 0.5	
hidden layer 3	dense, 512 neurons, ELU, Dropout 0.5	
output layer	dense, 1 neuron, Sigmoid activation	dense, 1 neuron, Sigmoid activation
regularization	L2 regularization (0.0001) on each Dense layer	none
optimizer	Adam, learning rate: 10^–3^	Adam, learning rate: 10^–4^
loss function	binary cross-entropy	binary cross-entropy

#### MVS-A

MVS-A was implemented using the MVS-A codebase.^13^ MVS-A uses an LGBMClassifier with 100 boosting iterations, lambda regularization of 1.0, column subsampling of 0.95, and a seed according to the current replicate number. The model was trained on the training set, and the self-importance scores were calculated using the following formula:
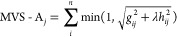
1with *g*_*ij*_ is the loss function gradient at checkpoint *i* for training sample *j* and *h*_*ij*_ is the Hessian at checkpoint *i* for training sample *j*.^13^

### Performance Metrics

In this study, we employed a suite of performance metrics to assess the effectiveness of our methods. The metrics used include top-*k* precision with *k* = 10, enrichment factor score at 10%, BEDROC score with an α parameter of 20,^42^ and average precision score. For statistical analysis, we applied the Wilcoxon signed-rank test with a significance level of α_W_ = 0.05,^35^ using the Benjamini–Hochberg correction for multiple method comparisons.^37,38^ For active learning and false and true positive prediction, a Friedman test was performed before data analysis to check for significant changes in distributions.^35,43^

## Data Availability

The code, the curated data set list, and the results published here are available on GitHub (https://github.com/JoshuaHesse/DataValuationPlatform). The system used for this study was configured with 256 GB of DDR4 3200 MHz RAM, an AMD Ryzen Threadripper 3970X 32-Core CPU, and two NVIDIA GTX 3070 GPUs.

## Supporting Information Available

Data curation section, statistical analyses, and additional experiments and discussion (PDF)
