# Supplementary material for: Machine Learning-Driven Data Valuation for Optimizing High-Throughput Screening Pipelines
Source: J Chem Inf Model. 2024 Oct 23;64(21):8142–52. doi: 10.1021/acs.jcim.4c01547 (PMC11558681; doi:10.1021/acs.jcim.4c01547)
Supplement: Supplementary file 1 — ci4c01547_si_001.pdf [file ci4c01547_si_001.pdf]

Supporting Information: Machine Learning-Driven Data Valuation for Optimizing High Throughput Screening Pipelines

Joshua Hesse, Davide Boldini,* and Stephan A. Sieber*

Technical University of Munich, TUM School of Natural Sciences, Department of Bioscience, Center for Functional Protein Assemblies (CPA), 85748 Garching bei München, Germany

E-mail: davide.boldini@tum.de; stephan.sieber@tum.de

Data Curation

Two different dataset groups were used in this study, one curated specifically for the false and true positive prediction and active learning experiment, and a well known benchmark for the undersampling project.

False Positive Detection and Active Learning

For our study on false positive detection and active learning, we selected 25 PubChem high-throughput screening (HTS) datasets, primarily based on the criteria set by the MF-PCBA benchmark.[S1] These datasets include both an initial high-throughput screen and a subsequent confirmatory screen, which focuses on a subset of the initially identified active

compounds. The false positive rates for these datasets are detailed in Table S1. A rate of 0 indicates that all initially active compounds were confirmed in the secondary screening, while a rate of 1 signifies that none of the primary actives were confirmed, indicating a 100% false positive rate. Detailed information regarding assay targets, AIDs, the nature of the HTS measurements, and the activity scores from the primary HTS screens is provided in Table S2. Corresponding data for the confirmatory screens are available in Table S3. Samples that were identified as active in the confirmatory screens according to the respective cutoffs (Table S3) were defined as true actives in the experiments, as they primary activity was confirmed in the confirmatory screen.

Table S1: Datasets used for false positive detection and active learning

**Name**	**Source**	**Number of compounds**	**FP rate**
transcription5	Buterez et al.[S1]	641690	0.86
transcription	Buterez et al.[S1]	344601	0.44
transporter	Butkiewicz et al.[S2]	306009	0.30
GPCR3	Butkiewicz et al.[S2]	63477	0.56
GPCR	Butkiewicz et al.[S2]	325567	0.51
hsf1	Buterez et al.[S1]	300776	0.75
ion_channel2	Butkiewicz et al.[S2]	104574	0.22
ion_channel3	Butkiewicz et al.[S2]	305283	0.35
ion_channel	Butkiewicz et al.[S2]	305185	0.16
methylation	Buterez et al.[S1]	309436	0.77
serine	Buterez et al.[S1]	213945	0.92
splicing	Buterez et al.[S1]	292971	0.12
streptokinase	Buterez et al.[S1]	302954	0.74
toxin_B	Buterez et al.[S1]	348764	0.96
ubiquitin	Buterez et al.[S1]	330076	0.71

Name	Source	Number of compounds	FP rate
zinc_finger	Buterez et al.[S1]	301422	0.49
fatty_acid_synthase	Buterez et al.[S1]	361599	0.04
RNA_binding	Buterez et al.[S1]	358765	0.99
transcription3	Buterez et al.[S1]	363339	0.83
transcription4	Buterez et al.[S1]	353506	0.07
transcription2	Buterez et al.[S1]	300881	0.79
GPCR2	Butkiewicz et al.[S2]	195949	0.84
cysteine_protease	Buterez et al.[S1]	344012	0.51
dna_repair	Buterez et al.[S1]	351707	0.37
channel_atp	Buterez et al.[S1]	343406	0.49

Table S2: Datasets primary assay details

Name	UniProt ID	HTS AID	HTS measurement	Activity score
GPCR	O43613	485270	Inhibition at 6.6 uM	PubChem activity score
GPCR_2	P25929	1040	Inhibition at 3.6 uM	PubChem activity score
GPCR_3	P08482	628	B score at 10 uM	B score at 10 uM
ion_channel	P35561	1672	B score at 10 uM	PubChem activity score
ion_channel_2	O95180	449739	B score at 10 uM	B score at 10 uM
ion_channel_3	O88943	2239	B score at 10 uM	PubChem activity score
transporter	Q9GZV3	488975	B score at 10 uM	PubChem activity score
transcription	CAD53472	504558	Activity at 12.5 uM	PubChem activity score
transcription_2	AAH94064	2098	Activity at 2.5 uM	PubChem activity score
transcription_3	P34707	624304	Inhibition at 21.8 uM	Ratio:Normalized
ubiquitin	P61088	485273	Inhibition at 20 uM	PubChem activity score
serine	Q9Y337	873	Inhibition at 5 uM	PubChem activity score

Name	UniProt ID	HTS AID	HTS measurement	Activity score
splicing	P9WHJ3	2221	Activity at 7.5 nM	PubChem activity score
channel_atp	P13569	720511	Activity at 7.5 uM	PubChem activity score
cysteine_protease	P55212	686996	Activity at 12.5 uM	PubChem activity score
zinc_finger	Q9XUB2	1832	Activity at 10 uM	PubChem activity score
DNA_Repair	P43351	651710	Inhibition at 18.71 uM	PubChem activity score
Toxin_B	Q189K3	652162	Inhibition at 9.99 uM	PubChem activity score
Streptokinase	P10520	1662	Inhibition at 7.5 uM	PubChem activity score
methylation	O96028	743445	Inhibition at 12.5 uM	PubChem activity score
Hsf1	Q52L52	504408	Activity at 9 uM	PubChem activity score
transcription_4	Q06710	652154	Activity at 12.62 uM	PubChem activity score
fatty_acid_synthase	P49327	602261	Inhibition at 15 uM	%Activity at 15 uM
RNA_binding	P11940	588489	Inhibition at 20 uM	%Activity at 20 uM corr
Transcription_5	O75030	1259374	inhibition at 2 uM	PubChem activity score

Table S3: Datasets confirmatory assay details

Name	UniProt ID	Assay AID	Assay measurement	Assay cutoff
GPCR	O43613	492964	2 Replicates	46.00%
GPCR_2	P25929	1254	2 Replicates	20.00%
GPCR_3	P08482	677	2 Replicates	3SD
ion_channel	P35561	2032	2 Replicates	3SD
ion_channel_2	O95180	489005	EC50	Failed fit
ion_channel_3	O88943	2287	2 Replicates	3SD
transporter	Q9GZV3	493221	2 Replicates	3SD
transcription	CAD53472	588343	AC50	350 uM or failed fit
transcription_2	AAH94064	2382	EC50	195 uM or failed fit

Name	UniProt ID	HTS AID	HTS measurement	Activity score
transcription_3	P34707	624474	IC50	Equation (pIC50, QC)
ubiquitin	P61088	493155	IC50	Equation (pIC50, QC)
serine	Q9Y337	1431	IC50	50 uM or failed fit
splicing	P9WHJ3	435010	EC50	380 uM or failed fit
channel_atp	P13569	743267	AC50	100 uM or failed fit
cysteine_protease	P55212	720632	AC50	70 uM or failed fit
zinc_finger	Q9XUB2	1960	EC50	300 uM or failed fit
DNA_Repair	P43351	652116	AC50	47 uM or failed fit
Toxin_B	Q189K3	720512	AC50	42 uM or failed fit
Streptokinase	P10520	1914	EC50	150 uM
methylation	O96028	1053173	AC50	20 uM or failed fit
Hsf1	Q52L52	435004	EC50	260 uM or failed fit
transcription_4	Q06710	687027	AC50	70 uM or failed fit
fatty_acid_synthase	P49327	624326	IC50	20 uM
RNA_binding	P11940	602259	IC50	20 uM
Transcription_5	O75030	1259375	IC50	5 uM

Importance Undersampling

In the undersampling application, we employed 10 datasets from the MolData benchmark, all associated with the disease *aging*.[S3] These *aging*-focused datasets vary in size, ranging from 195,624 to 408,917 compounds. The proportion of active to inactive compounds in these datasets spans from 8.7×10^{-5} to 9.3×10^{-3}. Collectively, these datasets encompass a total of 511,471 unique compounds, out of which 8,527 are classified as active. Details of these ten datasets are compiled in Table S4.

Table S4: MolData datasets of the disease group "aging"

Name	UniProt ID	Assay AID	Compounds	Actives
PTHR1	Q03431	743266	408917	308
Lamin_A	P02545	1487	198098	27
TNAP	P05186	1012	195634	518
PGC1_act	N/A	651723	349095	741
eLon	P0A9M0	602123	362345	1524
vitamin_receptor	P11473	504847	401152	3735
Pin1	Q13526	504891	387567	34
PGC1_inhib	N/A	651687	349095	603
TNAP_phos	P05186	1135	195624	66
HTRA	Q05DJ8	504803	343699	1713

Chemical Space Analysis

The diversity of hits across the different dataset collections was qualitatively assessed using Uniform Manifold Approximation and Projection (UMAP), with Extended-Connectivity Fingerprints (ECFPs) as the molecular representation, to visualize the broad coverage of the chemical space. Figure S1 illustrates the chemical diversity within the dataset collection curated for false positive prediction and active learning. Although this analysis includes only a subset of hits from ten out of the 25 datasets, it clearly demonstrates the substantial diversity of the compounds. Figure S1 B) highlights the diversity not only across the entire group of datasets but also within each individual dataset. The unsupervised UMAP does not cluster samples from separate datasets together, in contrast to the supervised UMAP in A). Figure S2 presents a similar analysis for the *aging* group from the MolData benchmark, revealing comparable diversity.

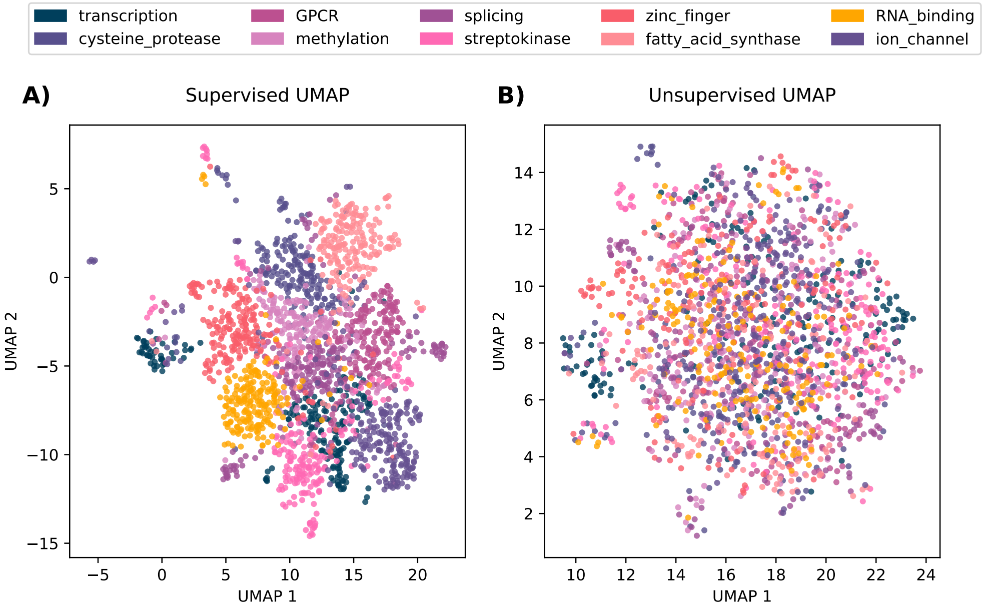

Figure S1: Chemical space covered by a subset of hits from the curated group of datasets: A) Supervised UMAP dimensionality reduction on ECFP representations of a subset of hits. B) Unsupervised UMAP dimensionality reduction on the same ECFPs.

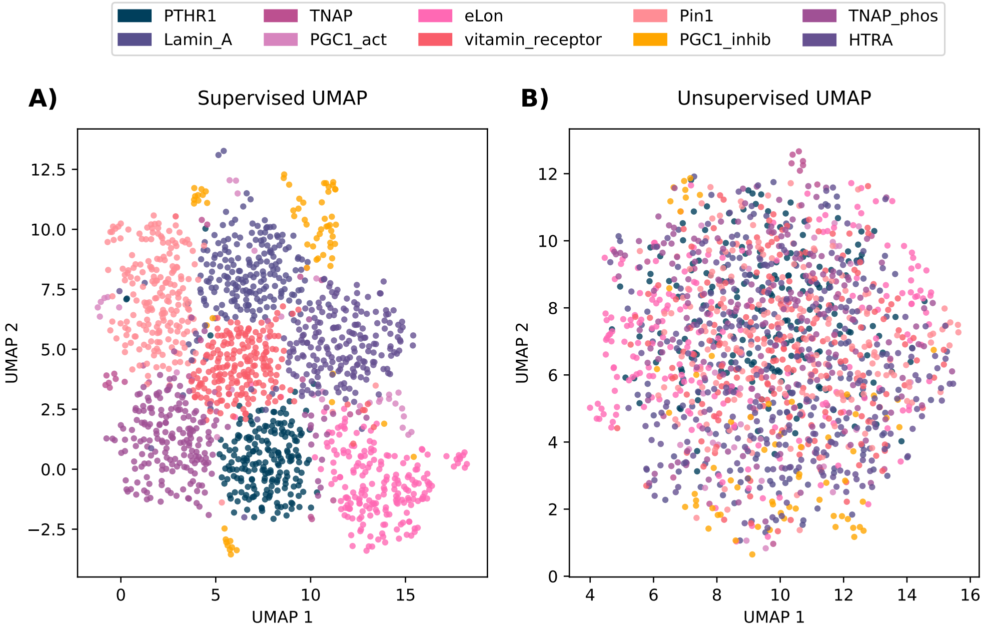

Figure S2: Chemical space covered by a subset of hits from the MolData aging group: A) Supervised UMAP dimensionality reduction on ECFP representations of a subset of hits. B) Unsupervised UMAP dimensionality reduction on the same ECFPs.

Importance Active Learning Optimization

During the development of the data valuation-based active learning pipeline, both the choice of regression function and the choice of sampling function were optimized on the *cysteine_protease* dataset.

Regression Functions

The following regression models were benchmarked:

LightGBM Regressor

Gradient boosting for regression was implemented using an LGBMRegressor with default settings. Regression was performed in five replicates at each step, recording both the average

predicted value and variance.

Support Vector Machine

The Support Vector Regressor (SVR) implementation from sklearn was used for regression via support vector machines, utilizing default parameters alongside a custom Tanimoto kernel for computing pairwise Tanimoto similarities. Due to memory constraints encountered while predicting the remaining training set samples during active learning, the process was performed in batches of 512 samples. This regression was replicated five times at each step, recording both the average predicted value and the variance.

Feedforward Neural Network

The Feedforward Neural Network (FNN) for regression was implemented as a TensorFlow sequential model with one hidden layer with 64 neurons and a Rectified Linear Unit (ReLU) activation function. The model's loss function was set to the mean absolute error and the Adam optimizer was used with a learning rate of 0.001. Training was performed over 50 epochs with a validation split of 0.2. The regression was done in five replicates per step, recording the average predicted value and variance.

Gaussian Process Regressor

Two Gaussian Process Regressor (GPR) implementations were benchmarked. The GPR GPflow implementation was used with the constant mean function, a noise variance of one, and a custom Tanimoto kernel, which calculates the Tanimoto similarities, originally implemented by the FlowMO package.[S4] This implementation uses the Graphics Processing Unit (GPU) for calculations, which resulted in out-of-memory issues. Therefore, the maximum

number of training samples was empirically set to 10,500. Similarly, prediction was done in batches of 512 samples to avoid memory issues. The predictions and variances were recorded. The sklearn GPR implementation was used as a faster and less memory-intense alternative, setting `alpha = 1` and using a custom Tanimoto kernel that adapted the FlowMO implementation to the sklearn library.[S4] Using this implementation without GPU usage, only the largest dataset (*transcription_5*) caused memory issues and was limited to 30,000 training samples. The predictions were conducted in the same manner as described for the GPflow implementation.

Tanimoto Kernel

As mentioned, SVR and GPR necessitate the use of kernels for handling high-dimensional data. These kernels are pivotal in such methodologies, offering a versatile and potent mechanism to assess similarity or distance between data points, which allows prediction of unseen samples by capturing complex connections in the data at hand.[S5]

The Tanimoto kernel, a prevalent similarity measure in cheminformatics and molecular modeling, is notably effective due to the widespread use of molecular fingerprints, especially ECFP vectors. This kernel excels in computing similarities on binary vectors, a task not feasible with measures like Euclidean distance. It calculates the Tanimoto similarity coefficient by dividing the intersection of two samples, for instance, two ECFP vectors, by their union. This coefficient is integral in GPR and SVR to enable the prediction of unseen samples.[S6]

Regression Function Optimization

Various regression models were tested in the importance active learning pipeline, always using Minimal Variance Sampling Analysis (MVS-A) for importance calculation, as MVS-A

has shown to have the best active learning performance. The original implementation using an LGBMRegressor performed equally to the greedy benchmark. Using a simple one-hidden layer deep neural network resulted in a similar performance.

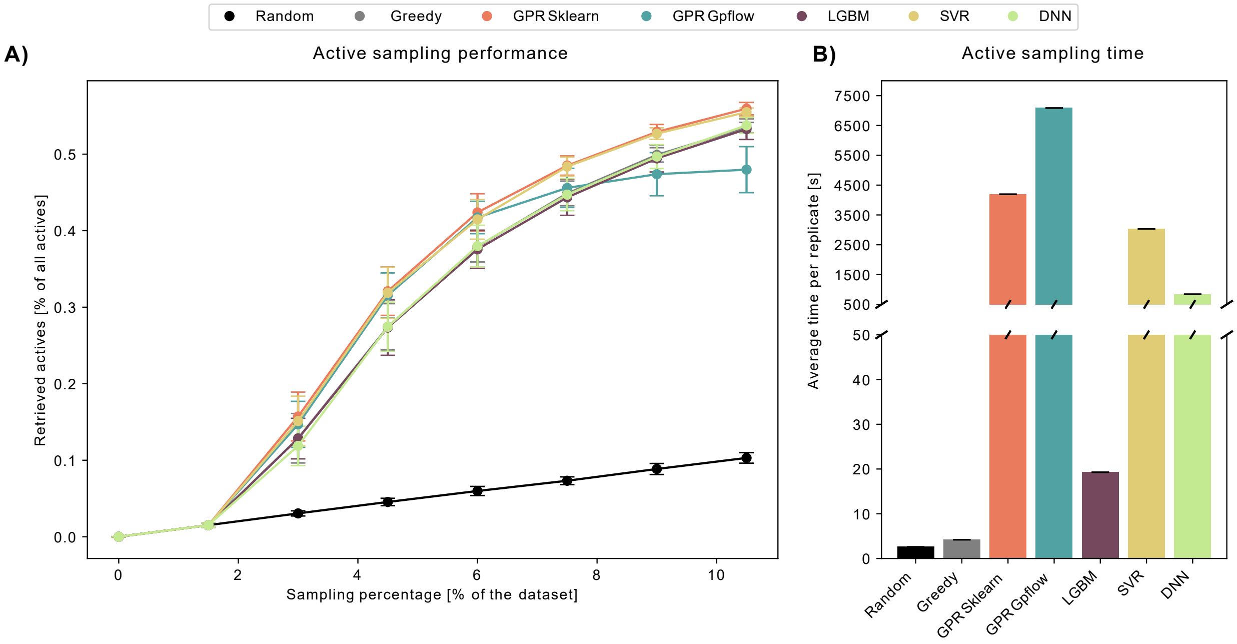

Figure S3: Active learning performance using different regression models: All calculations were done on the *cysteine_protease* dataset, with 10 replicates per method. Each step size, including the initial random sampling, is 1.5% of the entire dataset. A) Percentage of actives found after each step. A Friedman test shows a highly significant difference (p-value = 7.09e-10), indicating that the methods do not perform equally. B) Average time per replicate in seconds. P-value in the title refers to Friedman test on all methods.[S7]

A GPR is another model type adept at handling high-dimensional data. Its Bayesian framework enables it to compute uncertainty estimations, a topic explored further in section . Up to step 3, both GPR implementations, Gpflow and sklearn, performed comparably. However, at this stage, the Gpflow version, which utilizes GPU for training, encountered memory constraints due to the expanding size of the training set. Consequently, it was necessary to limit its training to the 10,500 most important samples. In contrast, the sklearn GPR implementation, operating on Central Processing Unit (CPU), did not face this limitation and maintained consistent performance beyond step 3. Additionally, it is noteworthy that while both GPR models demand significant computational resources, the sklearn implementation

is approximately 40% more efficient in terms of processing time.

The support vector regressor (SVR) showcased performance nearly on par with the sklearn GPR, while being more time-efficient. It is, however, crucial to note that this average time efficiency is in reference to replicates using the entire dataset. LightGBM, FNN, and SVR regressors were executed in five internal replicates for each prediction, providing both mean and variance. This approach was integral to determining the sampling strategy, which is elaborated on in the subsequent section. On the other hand, the GPR models were executed only once per prediction, as their design inherently facilitates immediate variance calculation. Thus, when considering individual iterations, the SVR actually achieves a substantial 85% time efficiency advantage over the sklearn GPR.

Although the sklearn GPR demands high computational time, it was selected for further analysis due to its marginally superior performance, which compensates for the longer calculation time. The absolute computation time of approximately 70 minutes for a medium-sized HTS library is deemed manageable. The sklearn GPR's ability to directly calculate uncertainty estimations proved particularly valuable for the final optimization phase, specifically in determining the most effective sampling strategy.

Sampling Functions

When screening previously unseen samples, such as a compound library, there is always a trade-off between exploitation and exploration. Exploitation describes the approach of taking actions that will result in the best performance judged on the current knowledge, such as targeting compounds as structurally similar to known actives, while exploration relates to actions that uncover previously unknown aspects, such as previously unknown domains of the chemical space.[S8]

One example, greedy sampling, targets the highest predicted scores, thereby being fully ex-

ploitative. A more explorative approach is Upper Confidence Bound (UCB) sampling, which introduces a parameter λ that allows tuning how much impact the variance of a prediction has on its acquisition.[S9] The larger λ is, the bigger the impact of a sample's variance is on its likelihood to be sampled, increasing the exploration at the cost of exploitation.

Sampling Function Optimization

To assess which sampling approach is more successful in this active learning setting, two sampling strategies were compared: the exploitative greedy sampling and the more explorative UCB sampling approach.

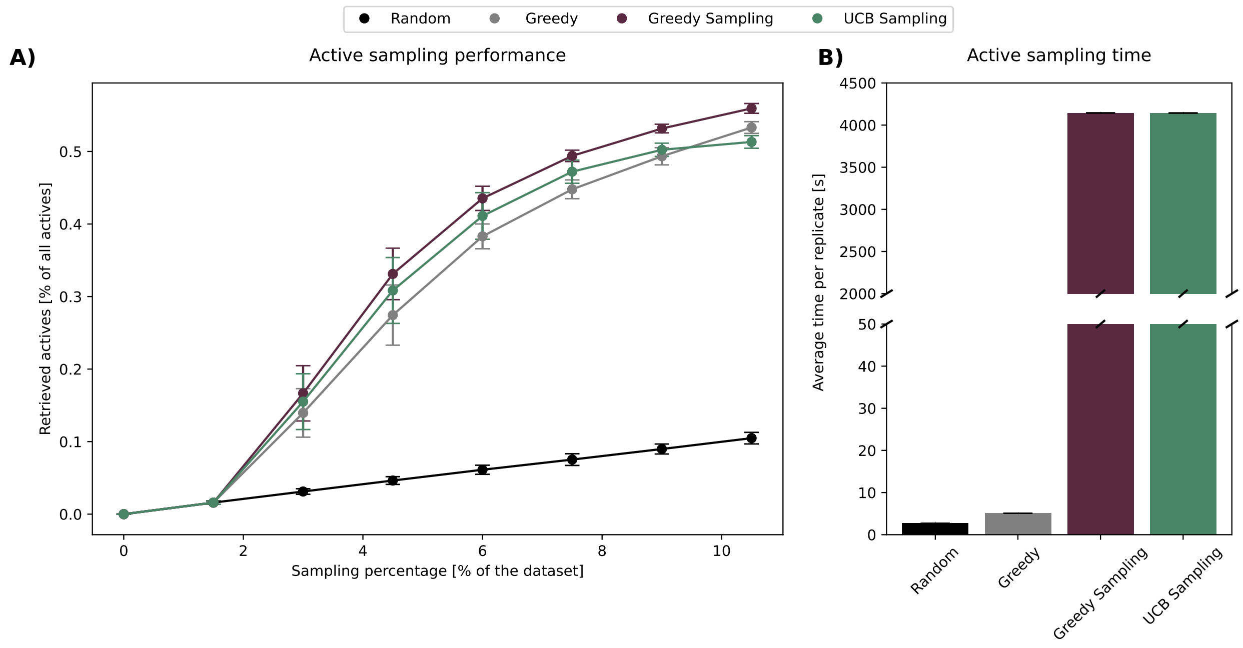

Figure S4: Active learning performance using different sampling strategies: All calculations were done on the *cysteine_protease* dataset, with 10 replicates per method. Each step size, including the initial random sampling, is 1.5% of the entire dataset. GPR sklearn was used for regression. For UCB sampling, λ was set to 2. A) Percentage of actives found after each step. B) Average time per dataset replicate in seconds.

Remarkably, the performance of the greedy sampling method surpasses that of the UCB method at every step. This outcome, albeit unexpected given the greedy method's em-

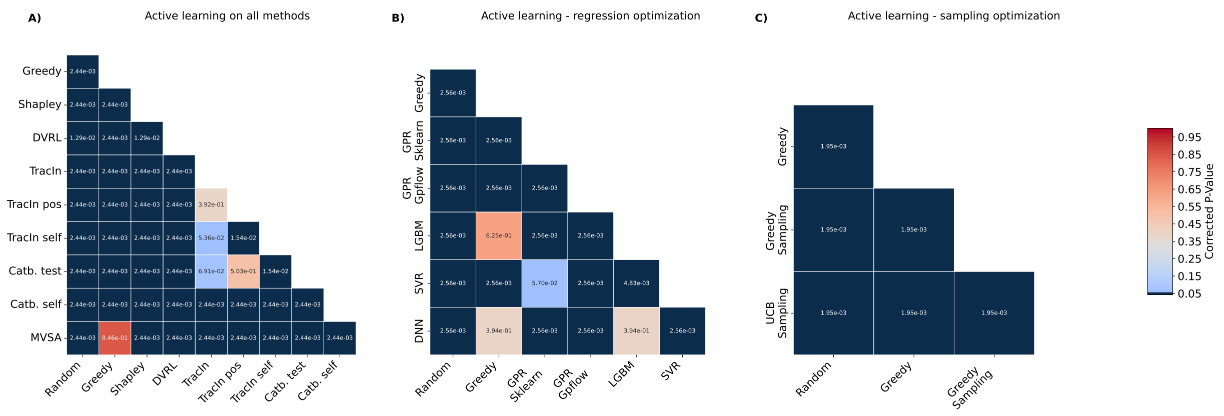

Figure S5: Significance analysis of active learning optimization experiments: Corrected p-values after Benjamini Hochberg correction using a Wilcoxon two-tailed single rank test averaged over 10 replicates at the final sampling step ($\alpha = 0.05$);[S7,S10,S11] all significant corrected p-values < 0.05 are marked in dark blue. A) Comparison of different data valuation methods. B) Comparison of different regression models in combination with MVSA. C) Comparison of different sampling methods in combination with MVSA and GPR SKlearn.

phasis on exploitation over exploration, aligns with prior findings by Graff et al. In their research, it was demonstrated that in a chemistry-focused active learning scenario, greedy sampling had an advantage over UCB sampling.[S9] A plausible explanation for the superior efficacy of greedy sampling compared to UCB lies in the non-exclusivity of exploitation and exploration in the context of self-importance-based sampling. As observed earlier, MVS-A attributes high scores to samples diverging from typical patterns. Consequently, greedy sampling targets compounds with elevated MVS-A predictions. This strategy, while exploitative in its targeting of the highest MVS-A scores, may inadvertently facilitate the investigation of a varied array of samples.

Scaffold Diversity in False and True Positive Predictions

The capability to identify a wide array of structural scaffolds is crucial for accurately detecting false positives, ensuring comprehensive identification across various types rather than being confined to particular subsets. This breadth is also vital in drug development during HTS for potential lead compounds, as a diverse array of true positives increases the probability of successful hit to lead optimization.[S12]

The scaffold diversity of both false and true positives, identified by the methods we previously introduced was assessed by calculating the proportion of unique Murcko scaffolds. These findings are detailed in Figure S6.

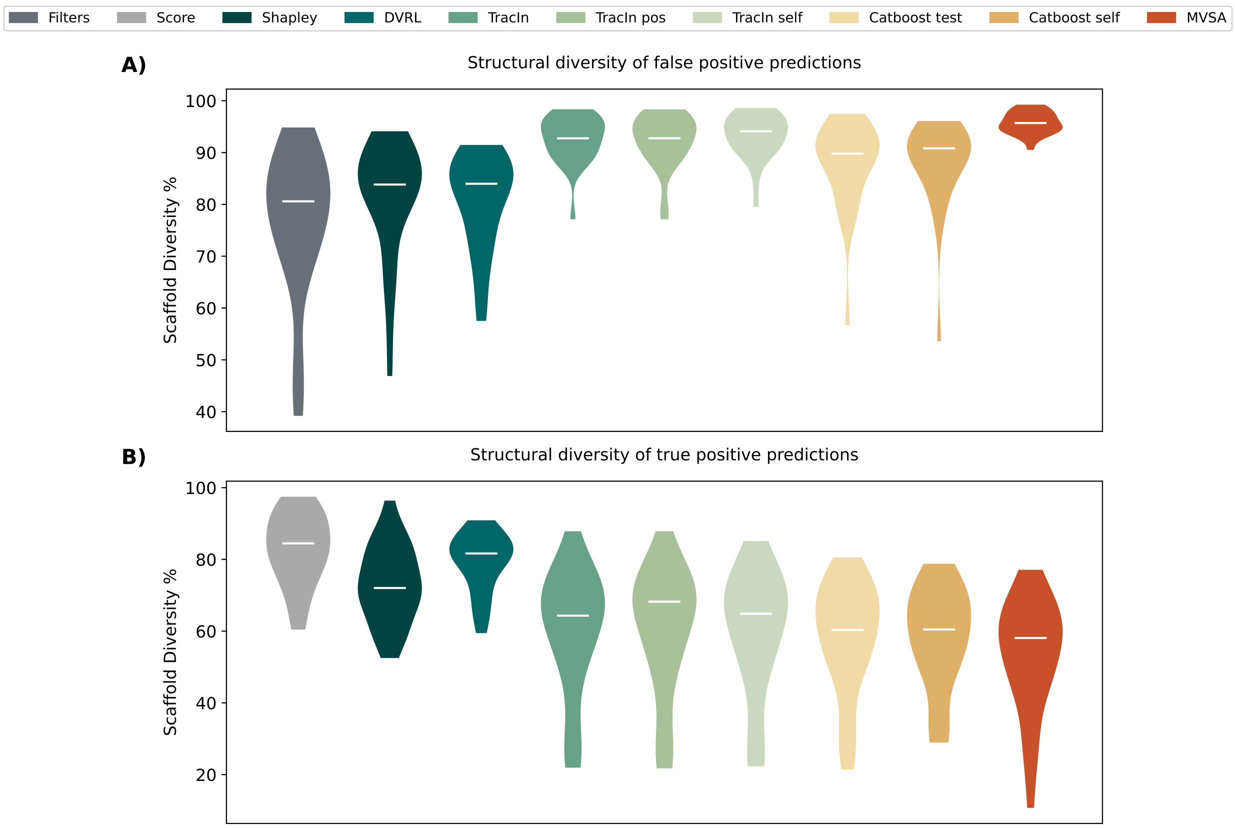

Figure S6: Murcko scaffold diversity in true and false positive hits: White bars indicate the median of the distribution. A) Distribution of Murcko scaffold diversity scores across all datasets for false positive detection. B) Distribution of Murcko scaffold diversity scores across all datasets for false positive detection.

In terms of false positive hit diversity, the GSK REOS filters benchmark showed a range of diversity from 60.6% to 100%, with a median value of 90.5%. However, all data valuation methods indicated higher median values, notably with MVS-A achieving a median of 94.8%, and a range from 90.5% to 99.2%, as depicted in Figure S6 A). Both MVS-A and Tracing Gradient Descent (TracIn) methods exhibited less variation in diversity, typically surpassing the benchmark median in most datasets. K-nearest neighbor (KNN) Shapley and Data Valuation using Reinforcement Learning (DVRL) showed scaffold diversities akin to the GSK REOS filters, suggesting that data valuation methods based on Gradient Boosting Machine (GBM) and FNN, particularly those utilizing self-importance, excel at identifying deviations from typical true positive patterns without predefined rules.

Self-importance scores are highest when a false positive's label cannot be predicted based on other actives, including other false positives. Therefore, the concept of self-importance-based false positive detection results in the identification of a diverse set of false positives. This, in turn, leads to a high level of false positive diversity across all datasets rather than being limited to specific domains. KNN Shapley, which spots samples clustering with inactives despite having an active label, tends to identify less diverse samples, as these false positives are identified not by their difference to other actives but by their similarity to other inactives. Combining methods like KNN Shapley and MVS-A could be beneficial, as they might offer complementary insights.

Conversely, true positive diversity shows an inverse pattern. The score benchmark demonstrated diversities between 60.4% and 97.5%, with a median of 84.5%. All importance-based methods, however, reported lower median values, with MVS-A recording the lowest at 58.1%. TracIn and CatBoost showed similar diversity distributions to MVS-A, while DVRL had a notably higher median true positive diversity at 81.7%. However, as observed in the main article, DVRL's seemingly random performance suggests that its measured diversity is a result of random sorting. KNN Shapley displayed relatively high scaffold diversities, which might

be due to its tendency to learn localized true positive patterns, unlike the broader patterns recognized by GBM and FNN models. MVS-A and TracIn define true positive samples as those whose activity is directly inferable from other active samples, inherently resulting in lower structural diversity compared to false positives.

In summary, gradient boosting models and FNNs tend to identify false positives by spotting deviations from learned patterns, yielding high diversity among false positives. Conversely, for true positive identification, these methods look for samples that closely match the learned patterns, leading to lower diversity in true positives. This contrasts with current benchmarks, where GSK REOS filters, limited by being structurally bound, captures a wider range of molecules.

False and True Positive Detection

Extended Connectivity Fingerprints

Additionally to the results shown in the manuscript for false and true positive prediction, the following figure shows the TP and FP predictions per Dataset.

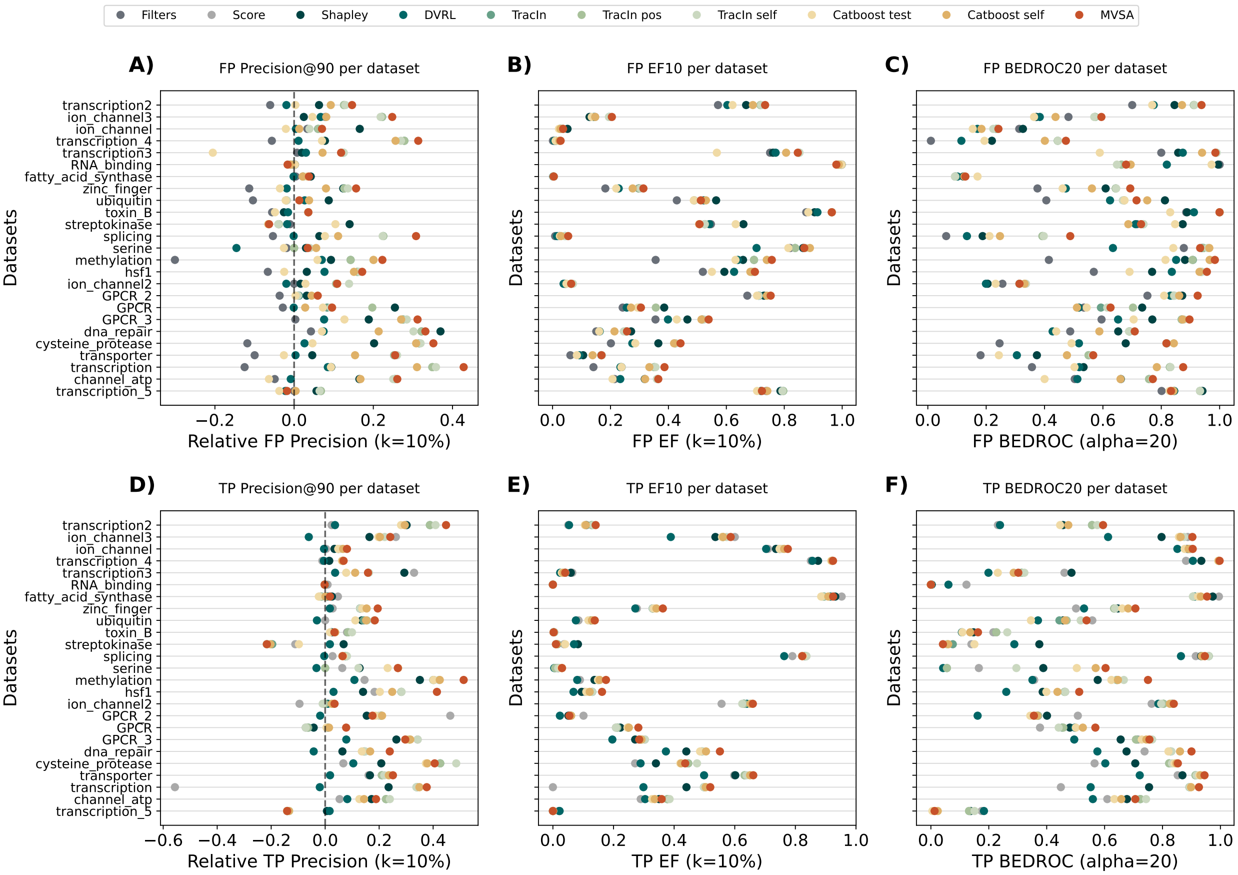

Figure S7: False positive detection performance across all datasets using ECFPs: A-C) False positive prediction performance per dataset. D-F) True positive prediction performance per dataset. A) Relative false positive precision per dataset. B) False positive enrichment factor per dataset. C) False positive Boltzmann-Enhanced Discrimination of the Receiver Operating Characteristic per dataset. D) Relative true positive precision per dataset. E) True positive enrichment factor per dataset. F) True positive Boltzmann-Enhanced Discrimination of the Receiver Operating Characteristic per dataset.

RDKit Descriptors

Additionally to using ECFPs, the false and true positive experiments were performed using RDKit descriptors.

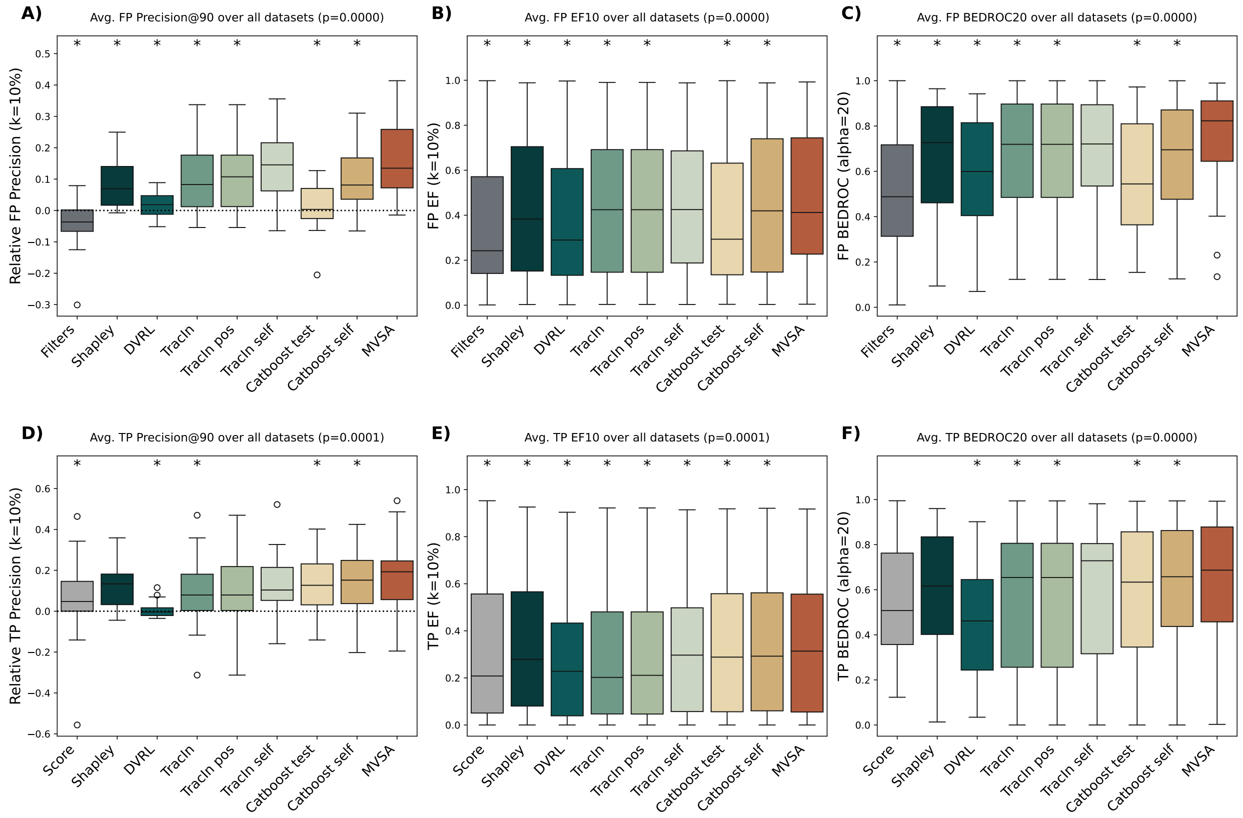

Figure S8: False positive detection performance across all datasets using RDKit descriptors: A-C) False positive performance metrices averaged over 25 datasets; asterisks indicate that the results are significantly different from MVSA ($\alpha < 0.05$, $n = 10$) according to a Wilcoxon two-tailed single rank test with Benjamini Hochberg correction.[S7,S10,S11] D-F) True positive performance metrices averaged over 25 datasets; asterisks indicate that the results are significantly different from MVSA ($\alpha < 0.05$, $n = 10$) according to a Wilcoxon two-tailed single rank test with Benjamini Hochberg correction.[S7,S10,S11] A) Relative false positive precision across all datasets. B) Mean false positive enrichment factor across all datasets. C) Mean false positive Boltzmann-Enhanced Discrimination of the Receiver Operating Characteristic across all datasets. D) Relative true positive precision across all datasets. E) Mean true positive enrichment factor across all datasets. F) Mean true positive Boltzmann-Enhanced Discrimination of the Receiver Operating Characteristic across all datasets. P-value in the title refers to Friedman test on all methods.[S7] All P-values are highly significant, indicating the methods do not perform equally according to all metrics presented.

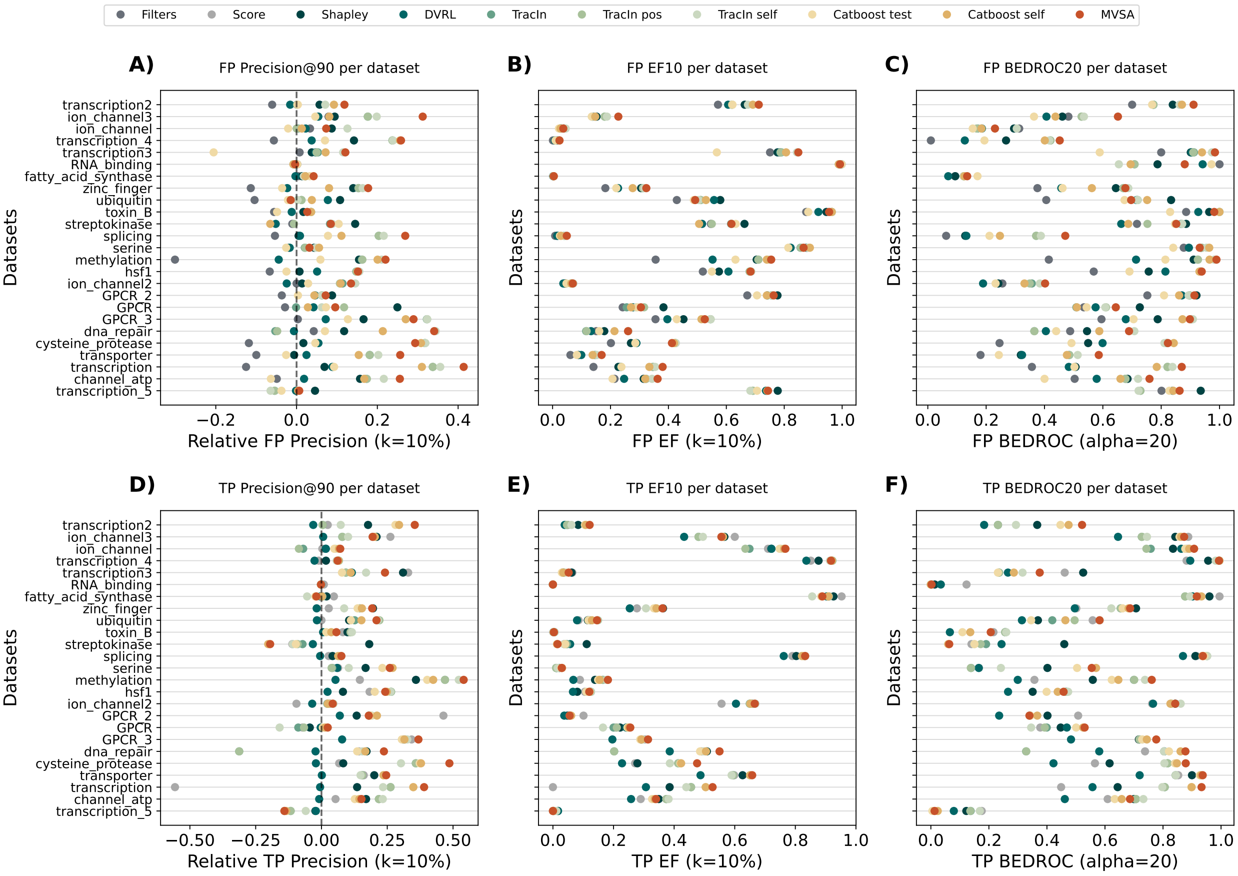

Figure S9: False positive detection performance across all datasets using RDKit descriptors: A-C) False positive prediction performance per dataset. D-F) True positive prediction performance per dataset. A) Relative false positive precision per dataset. B) False positive enrichment factor per dataset. C) False positive Boltzmann-Enhanced Discrimination of the Receiver Operating Characteristic per dataset. D) Relative true positive precision per dataset. E) True positive enrichment factor per dataset. F) True positive Boltzmann-Enhanced Discrimination of the Receiver Operating Characteristic per dataset.

TracIn on SMILES Strings

TracIn was adapted to using string-based input by using an LSTM. The false and true positive prediction experiments were then performed using SMILES strings as input features.

Computational Cost Evaluation

All data valuation approaches were timed on the false positive detection application on all 25 datasets using ECFPs.The average time per replicate over all datasets and for the individual datasets were recorded and are shown in Figure S12.

Statistical Tests

For the false and true positive prediction performances, all method results were tested for significance against each other within each molecular representation. A Wilcoxon two-tailed single rank test with Benjamini Hochberg correction with $\alpha = 0.05$ and 10 replicates was applied.[S7,S10,S11] The results for each of the molecular representations are shown in the following:

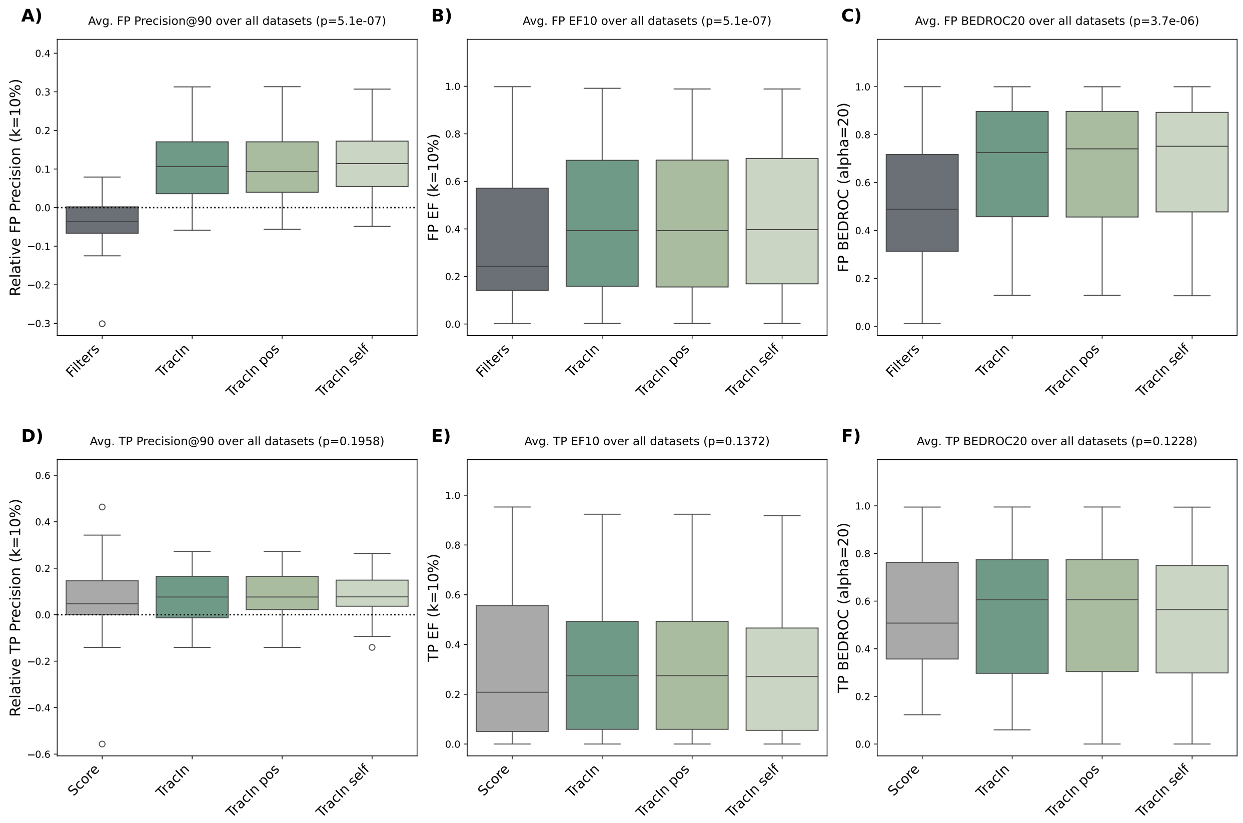

Figure S10: False positive detection performance across all datasets using TracIn on SMILES: A-C) False positive performance metrices averaged over 25 datasets; asterisks indicate that the results are significantly different from MVSA ($\alpha < 0.05$, $n = 10$) according to a Wilcoxon two-tailed single rank test with Benjamini Hochberg correction.[S7,S10,S11] D-F) True positive performance metrices averaged over 25 datasets; asterisks indicate that the results are significantly different from MVSA ($\alpha < 0.05$, $n = 10$) according to a Wilcoxon two-tailed single rank test with Benjamini Hochberg correction.[S7,S10,S11] A) Relative false positive precision across all datasets. B) Mean false positive enrichment factor across all datasets. C) Mean false positive Boltzmann-Enhanced Discrimination of the Receiver Operating Characteristic across all datasets. D) Relative true positive precision across all datasets. E) Mean true positive enrichment factor across all datasets. F) Mean true positive Boltzmann-Enhanced Discrimination of the Receiver Operating Characteristic across all datasets. P-value in the title refers to Friedman test on all methods.[S7] The P-values for false positive detection are significant, suggesting the methods do not perform equally. The P-values for true positive detection suggest the methods do not perform significantly different given the application.

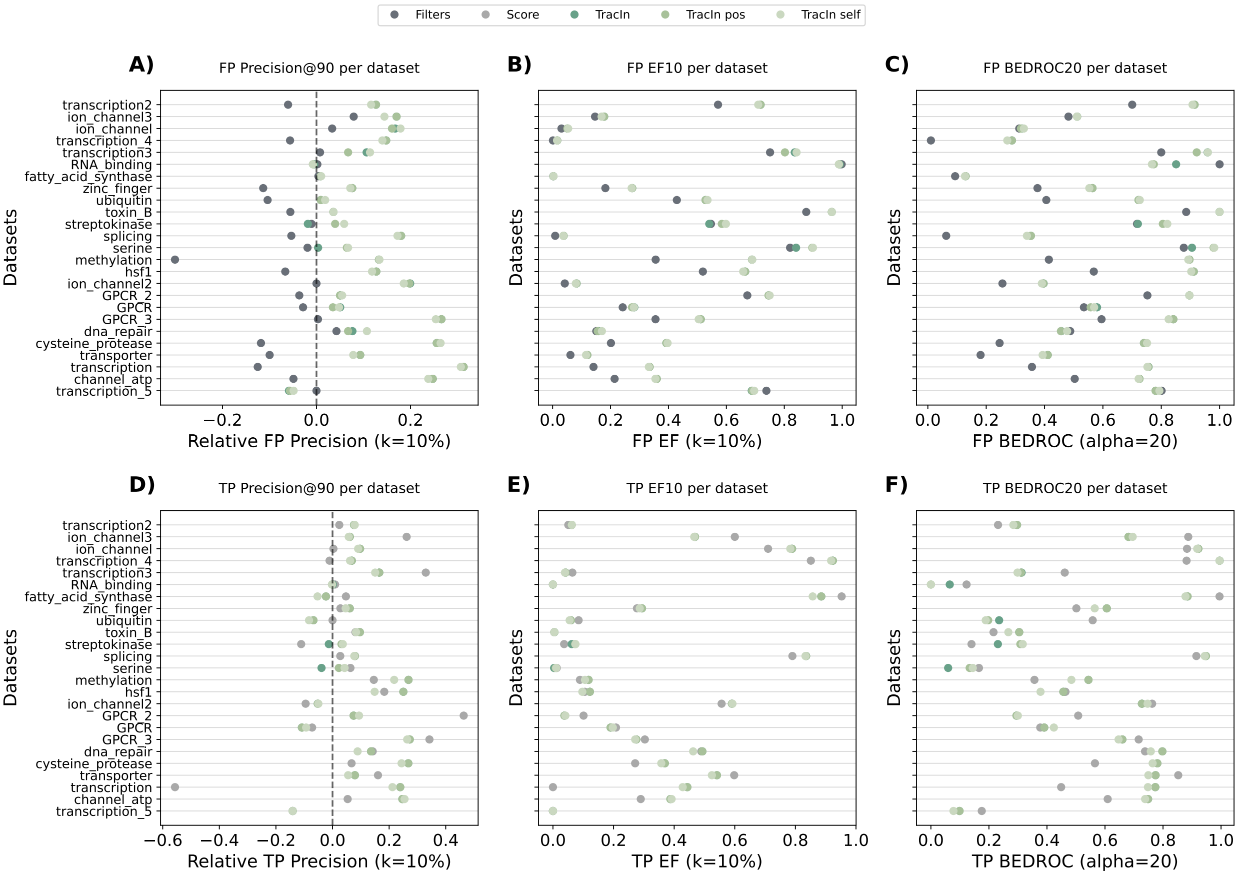

Figure S11: False positive detection performance across all datasets using TracIn on SMILES: A-C) False positive prediction performance per dataset. D-F) True positive prediction performance per dataset. A) Relative false positive precision per dataset. B) False positive enrichment factor per dataset. C) False positive Boltzmann-Enhanced Discrimination of the Receiver Operating Characteristic per dataset. D) Relative true positive precision per dataset. E) True positive enrichment factor per dataset. F) True positive Boltzmann-Enhanced Discrimination of the Receiver Operating Characteristic per dataset.

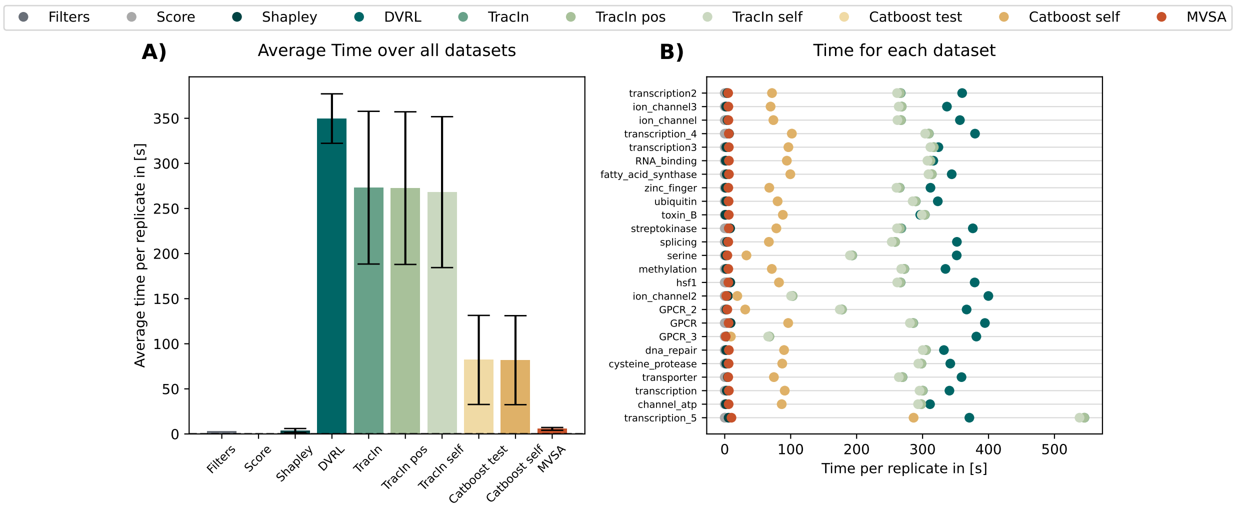

Figure S12: Computation time comparison: A) Average computation time per replicate in seconds across all datasets for all data valuation methods and benchmark approaches. B) Computation time per method and dataset. Data was generated while using ECFPs.

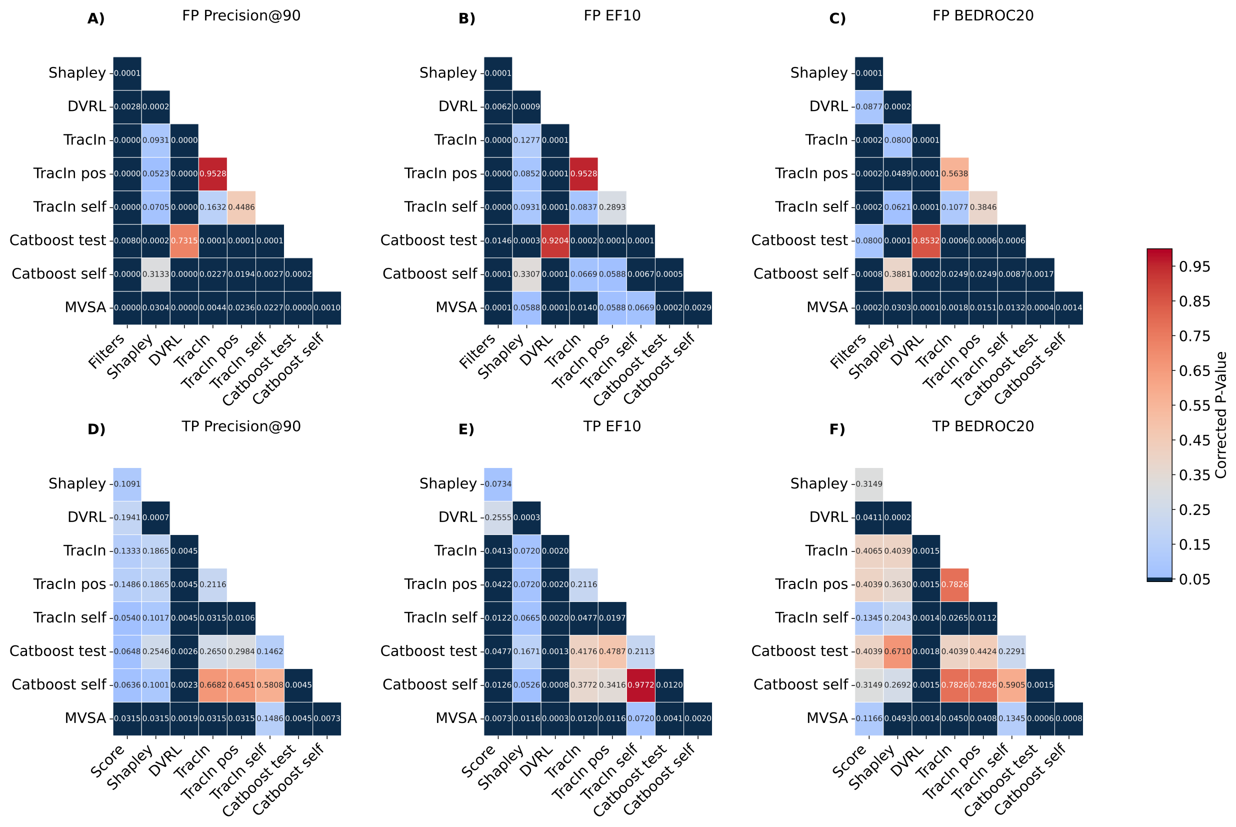

Figure S13: Significance analysis of false and true positive predictions using ECFPs: Corrected p-values after Benjamini Hochberg correction using a Wilcoxon two-tailed single rank test averaged over 25 datasets ($\alpha < 0.05$, $n = 10$);[S7,S10,S11] all significant corrected p-values < 0.05 are marked in dark blue. A-C) Corrected p-values for false positive prediction metrics. D-F) Corrected p-values for true positive prediction metrics.

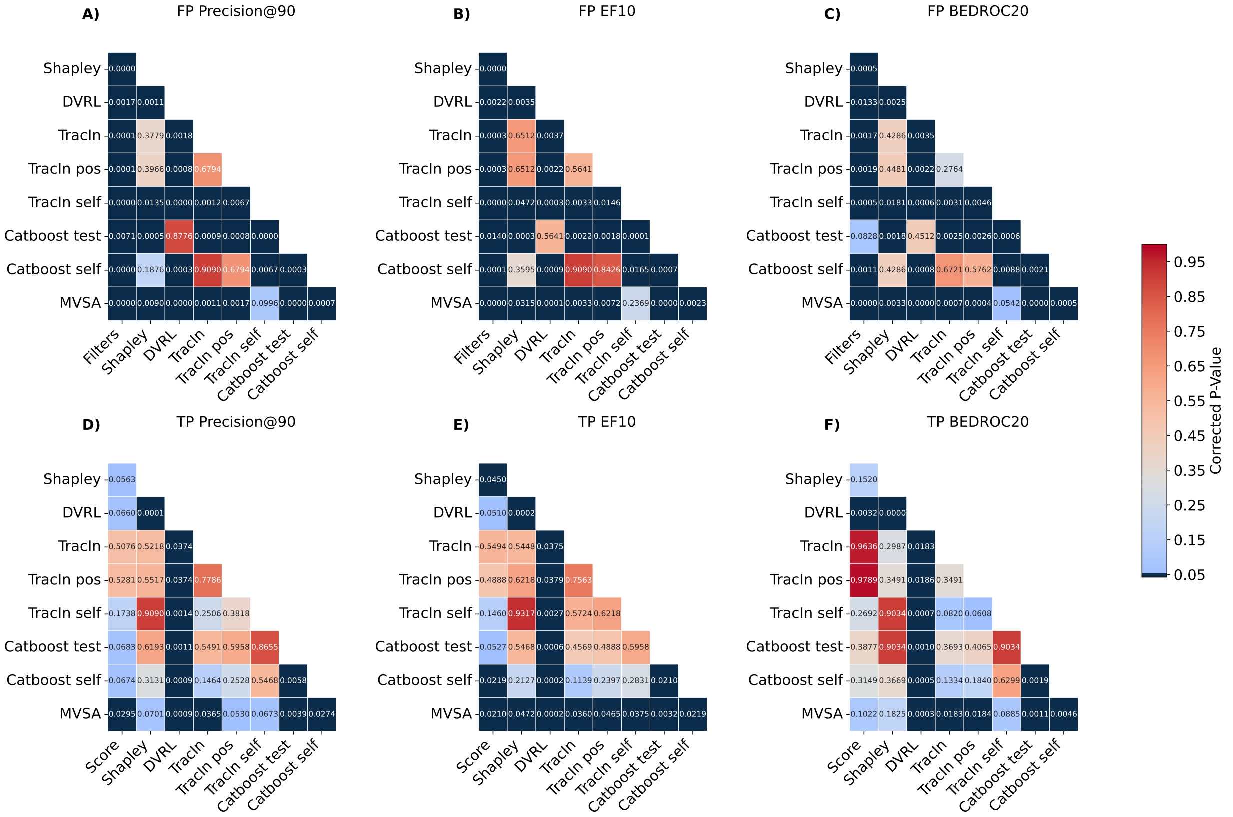

Figure S14: Significance analysis of false and true positive predictions using RDKit descriptors: Corrected p-values after Benjamini Hochberg correction using a Wilcoxon two-tailed single rank test averaged over 25 datasets ($\alpha < 0.05$, $n = 10$);[S7,S10,S11] all significant corrected p-values < 0.05 are marked in dark blue. A-C) Corrected p-values for false positive prediction metrics. D-F) Corrected p-values for true positive prediction metrics.

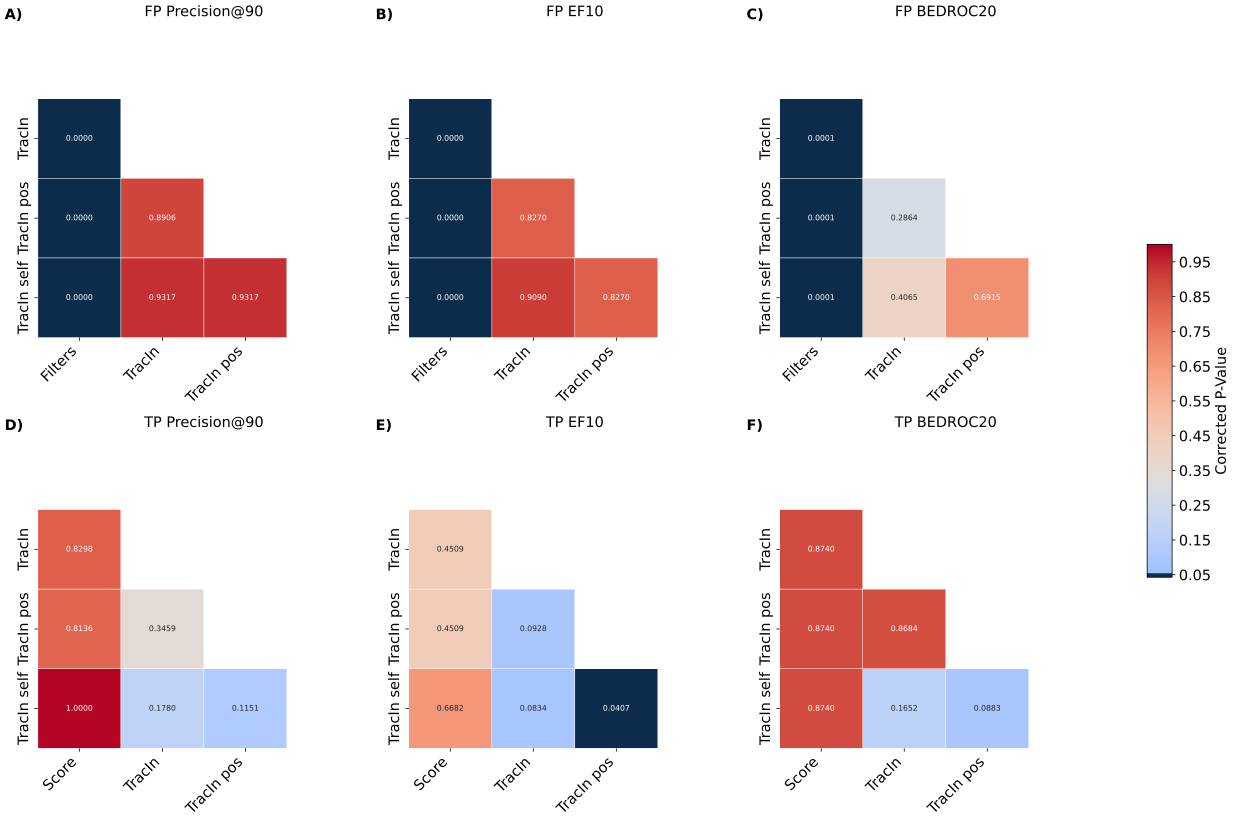

Figure S15: Significance analysis of false and true positive predictions using Simplified Molecular Input Line Entry System: Corrected p-values after Benjamini Hochberg correction using a Wilcoxon two-tailed single rank test averaged over 25 datasets ($\alpha < 0.05$, $n = 10$);[S7,S10,S11] all significant corrected p-values < 0.05 are marked in dark blue. A-C) Corrected p-values for false positive prediction metrics. D-F) Corrected p-values for true positive prediction metrics.

Undersampling

The results from main fig. 6 only encompass 4 out of 10 datasets in the aging dataset group. The experiment was also performed on the entire group. However, for the remaining 6 datasets the precision using a standard LightGBM model is far below 10% as seen in Table S5. The results can be seen in Figure S16. The shift between importance-based and random-based sampling is lower compared to main fig. 6, as importance scores become uninformative when a model's performance is random.

Table S5: LGBM precision on moldata disease group "aging" datasets

Name	Assay AID	Precision
PTHR1	743266	0.001
vitamin_receptor	504847	0.174
Lamin_A	1487	0.001
Pin1	504891	0.012
TNAP	1012	0.285
PGC1_inhib	651687	0.034
PGC1_act	651723	0.028
TNAP_phos	1135	0.167
eLon	602123	0.074
HTRA	504803	0.194

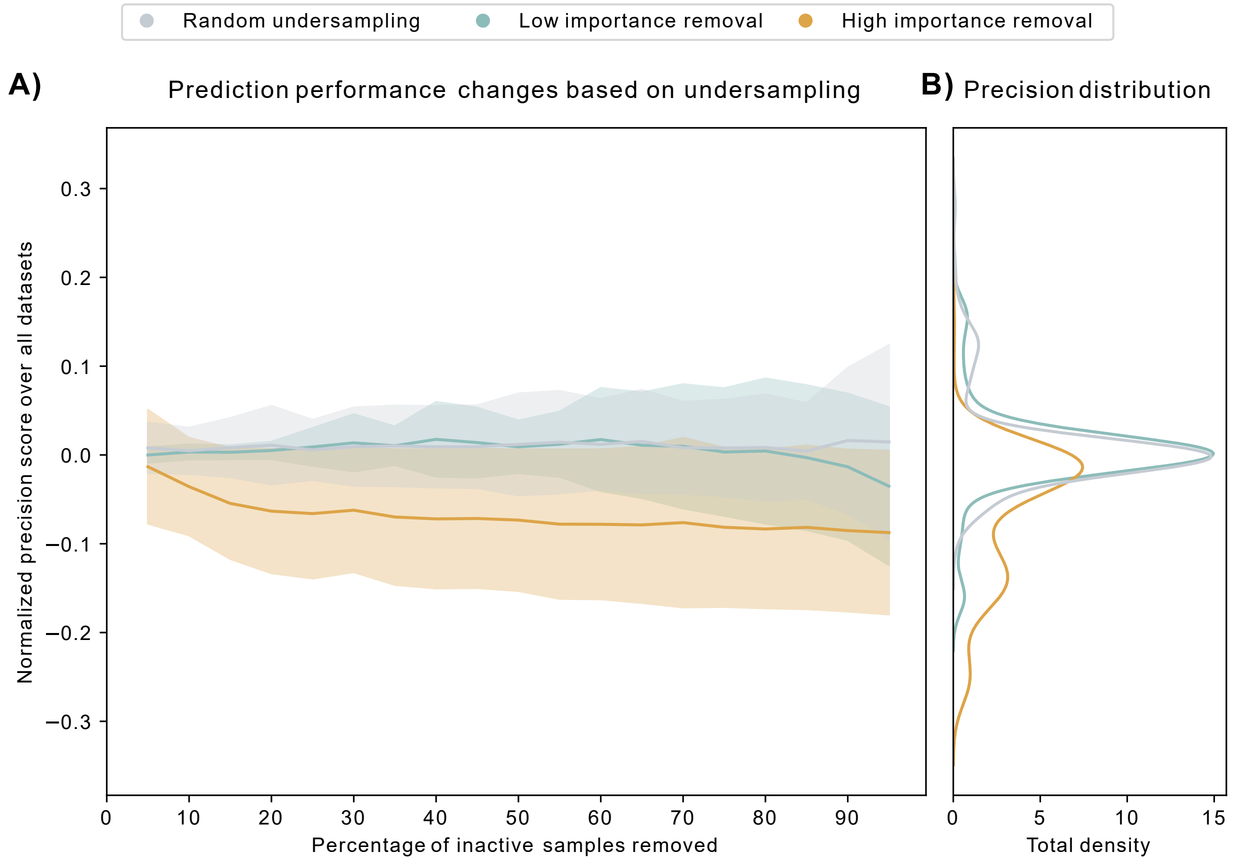

Figure S16: Significance analysis of false and true positive predictions using Simplified Molecular Input Line Entry System: Corrected p-values after Benjamini Hochberg correction using a Wilcoxon two-tailed single rank test averaged over 25 datasets ($\alpha < 0.05$, $n = 10$);[S7,S10,S11] all significant corrected p-values < 0.05 are marked in dark blue. A-C) Corrected p-values for false positive prediction metrics. D-F) Corrected p-values for true positive prediction metrics.

References

(S1) Buterez, D.; Janet, J. P.; Kiddle, S. J.; Liò, P. MF-PCBA: Multifidelity High-Throughput Screening Benchmarks for Drug Discovery and Machine Learning. *Journal of Chemical Information and Modeling* **2023**, *63*, 2667–2678.

(S2) Butkiewicz, M.; Wang, Y.; Bryant, S. H.; Lowe, E. W. J.; Weaver, D. C.; Meiler, J. High-Throughput Screening Assay Datasets from the PubChem Database. *Chemical informatics (Wilmington, Del.)* **2017**, *3*.

(S3) Keshavarzi Arshadi, A.; Salem, M.; Firouzbakht, A.; Yuan, J. S. MolData, a molecular benchmark for disease and target based machine learning. *Journal of Cheminformatics* **2022**, *14*, 10.

(S4) Matthews, A. G. d. G.; van der Wilk, M.; Nickson, T.; Fujii, K.; Boukouvalas, A.; León-Villagrá, P.; Ghahramani, Z.; Hensman, J. GPflow: A Gaussian process library using TensorFlow. *Journal of Machine Learning Research* **2017**, *18*, 1–6.

(S5) Ben-Hur, A.; Ong, C. S.; Sonnenburg, S.; Schölkopf, B.; Rätsch, G. Support Vector Machines and Kernels for Computational Biology. *PLOS Computational Biology* **2008**, *4*, e1000173.

(S6) Willett, P. The Calculation of Molecular Structural Similarity: Principles and Practice. *Molecular Informatics* **2014**, *33*, 403–413.

(S7) Virtanen, P.; Gommers, R.; Oliphant, T. E.; Haberland, M.; Reddy, T.; Cournapeau, D.; Burovski, E.; Peterson, P.; Weckesser, W.; Bright, J. et al. SciPy 1.0: Fundamental Algorithms for Scientific Computing in Python. *Nature Methods* **2020**, *17*, 261–272.

(S8) Dearden, R.; Friedman, N.; Andre, D. Model-Based Bayesian Exploration. 2013.

(S9) Graff, D. E.; Shakhnovich, E. I.; Coley, C. W. Accelerating high-throughput virtual screening through molecular pool-based active learning. *Chemical Science* **2021**, *12*, 7866–7881.

(S10) Benjamini, Y.; Hochberg, Y. Controlling the False Discovery Rate: A Practical and Powerful Approach to Multiple Testing. *Journal of the Royal Statistical Society: Series B (Methodological)* **2018**, *57*, 289–300.

(S11) Seabold, S.; Perktold, J. statsmodels: Econometric and statistical modeling with python. 9th Python in Science Conference. 2010.

(S12) Hoffer, L.; Muller, C.; Roche, P.; Morelli, X. Chemistry-driven Hit-to-lead Optimization Guided by Structure-based Approaches. *Molecular Informatics* **2018**, *37*.
